# Umbilical Cord Mesenchymal Stromal Cells for Cartilage Regeneration Applications

**DOI:** 10.1155/2022/2454168

**Published:** 2022-01-06

**Authors:** E. Russo, M. Caprnda, P. Kruzliak, P. G. Conaldi, C. V. Borlongan, G. La Rocca

**Affiliations:** ^1^Section of Histology and Embryology, Department of Biomedicine, Neurosciences and Advanced Diagnostics, University of Palermo, Italy; ^2^1st Department of Internal Medicine, Faculty of Medicine, Comenius University and University Hospital, Bratislava, Slovakia; ^3^2nd Department of Surgery, Faculty of Medicine, Masaryk University and St. Anne's University Hospital, Brno, Czech Republic; ^4^Department of Research, IRCCS ISMETT—Mediterranean Institute for Transplantation and Advanced Specialized Therapies, Palermo, Italy; ^5^Department of Neurosurgery and Brain Repair, Morsani College of Medicine, University of South Florida, Tampa, FL, USA

## Abstract

Chondropathies are increasing worldwide, but effective treatments are currently lacking. Mesenchymal stromal cell (MSCs) transplantation represents a promising approach to counteract the degenerative and inflammatory environment characterizing those pathologies, such as osteoarthritis (OA) and rheumatoid arthritis (RA). Umbilical cord- (UC-) MSCs gained increasing interest due to their multilineage differentiation potential, immunomodulatory, and anti-inflammatory properties as well as higher proliferation rates, abundant supply along with no risks for the donor compared to adult MSCs. In addition, UC-MSCs are physiologically adapted to survive in an ischemic and nutrient-poor environment as well as to produce an extracellular matrix (ECM) similar to that of the cartilage. All these characteristics make UC-MSCs a pivotal source for a stem cell-based treatment of chondropathies. In this review, the regenerative potential of UC-MSCs for the treatment of cartilage diseases will be discussed focusing on in vitro, in vivo, and clinical studies.

## 1. Introduction

Chondropathies are a group of cartilage diseases that deviate from or interrupt the normal structure and function of cartilage, including osteoarthritis (OA), achondroplasia, spinal disc herniation (SDH), relapsing polychondritis, cartilage tumor (CT), and chondrocalcinosis [1]. There are over 100 types of arthritis. The most common forms are OA (degenerative joint disease) and rheumatoid arthritis (RA, autoimmune form of arthritis). OA is the most well-known chondropathy in the world, affecting the health of 343 million of people, while RA affects 14 million of people [2]. OA is a multifactorial and complex degenerative joint disease characterized by age-related “wear and tear,” chondrocytes' poor response to growth factors, altered biomechanical properties of articular cartilage, mitochondrial dysfunction, oxidative stress, and inflammation [3]. The degenerative lesions in cartilage are secondary to inflammation associated with hyperplasia and chondrocyte apoptosis [4]. Increasing age is linked to a reduction in subchondral blood vessels resulting in cartilage-related physiological and biochemical anomalies [5]. This pathological process results in secondary joint fibrosis, stiffness, pain, and decreased function, leading to a poor quality of life. Risk factors for chondropathies include trauma, genetics, age, sex, obesity, and degenerative pathology. The biological mechanisms of chondropathies remain largely unknown, and there is no effective way to treat the cartilage damage because of its nature.

Cartilage is a supportive connective tissue, and it has a dense and highly organized extracellular matrix (ECM) embedding chondrocytes [6]. Three types of cartilage tissue are present throughout the body at various sites: hyaline, elastic, and fibrocartilaginous. Hyaline cartilage is the predominant form of cartilage and is present on the articular surfaces of synovial joints. Type II collagen is the main component in healthy articular cartilage. Other collagens of cartilage ECM are types III, VI, IX, X, XI, XII, and XIV. The main proteoglycan present in cartilage is aggrecan, but other proteoglycans found in cartilage include the syndecans, glypican, decorin, biglycan, fibromodulin, lumican, epiphycan, and perlecan. The chondrocytes are contained in cavities called lacunae embedded in the network of collagen fibrils and proteoglycans [6].

Cartilage has a decreased ability to self-repair because is avascular, resulting in a poor replicative capacity of chondrocytes. The lack of vascularity, along with the dense packing of ECM components, hinders the transport of drugs in the tissue, thus, challenging the treatments of cartilage diseases. In addition, cartilage also lacks nerve cells or endings and, therefore, cannot directly generate pain that is the main symptom of a chondropathy [7]. Therefore, symptoms usually occur only after the significant structural destruction of the cartilage ECM (with the damage affecting other tissues of the whole joint that do contain nociceptors), thus, making treatments difficult.

Current treatment of articular cartilage defects includes pharmacological management of pain, weight reduction, and exercises as well as intra-articular treatments with corticosteroid or hyaluronan and hylan derivatives injections [8]. Surgical options consist in bone marrow stimulation procedures such as subchondral drilling, microfracture, and abrasion arthroplasty, allowing endogenous mesenchymal stem cells (MSCs) to migrate into the lesion [9]. However, no treatment or procedure represents a cure for cartilaginous defects.

MSC-based therapy is beginning to show great potential in cartilage regeneration through several mechanisms including homing, angiogenesis, differentiation, and response to inflammatory condition. The most widely studied sources of MSCs are bone marrow (BM) and adipose tissue (AT). However, umbilical cord-derived MSCs (UC-MSCs) compared to AT- and BM-MSCs have many advantages such as higher proliferation rates, greater expansion ability, higher purity, and abundant supply along with no risks for the donor, since the UC is usually discarded after birth [10]. In Table 1 are listed the advantages and disadvantages of the different populations of stem/stromal cells used so far for cartilage regeneration purposes. UC-MSCs can be isolated from different regions of the UC stromal tissue called Wharton's Jelly (WJ), and three different populations of UC-MSCs have been obtained: perivascular (PV-MSCs), intermediate WJ (WJ-MSCs), and subamniotic stromal region or cord lining (CL-MSCs) [11]. Notably, ECM components in WJ share several features with cartilage ECM. To this regard, UC-MSCs express aggrecan, type II collagen, and SOX-9 [12]. In addition, UC-MSCs express growth factors, chemokines, and cytokines at similar levels to those of cartilage [12]. Finally, since UC relies on only two arteries and one vein to supply oxygen and nutrients, without any capillary circulation, UC-MSCs are physiologically adapted to survive in a relatively hypoxic environment leading to the potential advantage to survive in cartilage. These results reinforce the concept of UC-MSCs as one of the best candidates for MSC-based therapy for cartilage regeneration.

## 2. Regeneration Mechanisms of UC-MSCs for Cartilage Diseases

There are two main concepts for UC-MSCs' contribution to cartilage repair: preventing the degradation of cartilage, through the secretion of bioactive factors, and/or the differentiative potential of UC-MSCs to become chondrocytes. Several *in vitro* and *in vivo* studies indicated that UC-MSCs can play crucial roles in cartilage repair and regeneration by several mechanisms including (i) migration and homing, (ii) adaptation to cartilage hypoxic and nutrient-poor environment, (iii) chondrogenesis differentiation potential and promotion of survival, proliferation and differentiation of endogenous MSCs, (iv) synthesis and prevention of cartilage ECM degradation, and (v) anti-inflammatory and immunomodulatory properties.

### 2.1. Homing and Migration

MSCs exhibit certain capabilities of the homing of local mature leukocytes to inflammatory sites, such as rolling and adhesion [13]. Migration and homing ability into injured sites are considered as the primary steps for tissue repair in regenerative medicine. Different molecules mediate cell retention or mobilization such as adhesion molecules (E and P-selectin), integrins (particularly *α*4*β*1), stromal-derived factor 1 (SDF-1), and its receptor CXC chemokine receptor 4 (CXCR4) [13]. In addition, other factors play a key role in damaged joints homing such as CXCR1 and CXCR2, CC chemokine receptor 1 (CCR1), and monocyte chemoattractant protein 1 (MCP-1) through its receptor CCR2, vascular endothelial growth factor receptor 1 (Flt-1), platelet-derived growth factor receptor *α* (PDGFR-*α*, CD140a), PDGFR-*β* (CD140b), and their respective ligands IL-8, macrophage inflammatory protein 1*α* (MIP-1*α*), placenta growth factor (PlGF), and PDGF [14]. In Table 2, we reported different proteins involved in homing and migration of UC-MSCs in comparison with those expressed by BM- and AT-MSCs. In particular, BM- and AT-MSCs show similar expression pattern. On the other hand, based on the quantitative data reported in literature, UC-MSCs express higher levels of different proteins than BM- and AT-MSCs such as HGF, IL-8, IL-1RA, IGF-1, ICAM-1, bFGF, MCP-1, MIP-1*β*, PDGF-AA, PDGF-AB, PDGF-R, CXCR4, CCR2, VEGF-A, and VEGF-1. Interestingly, unlike cells from BM- and AT-MSCs, UC-MSCs express integrin *α*4 and MIP-1*β* suggesting their strong ability in homing and migration. In addition, UC-MSCs show less hematopoietic effects than BM- and AT-MSCs (low levels of SDF-1 and VCAM-1).

Increased levels of SDF-1, MCP-1, IL-8, MIP-1 *α*, PlGF, and PDGF were found in synovial fluid of OA and RA patients [14]. Shen et al. demonstrated that UC-MSCs secrete growth factors and chemokines which may contribute to a chemoattractive environment such as SDF-1, MCP-1, hepatocyte growth factor (HGF), vascular cell adhesion protein-1 (VCAM-1), IL-8, insulin-like growth factor-1 (IGF-1), and vascular endothelial growth factor (VEGF) [15]. In addition, UC-MSCs express CXCR4, CCR2, and c-met receptors. Therefore, UC-MSCs are able to migrate *in vitro* and *in vivo* via the SDF-1/CXCR4 and MCP-1/CCR2 axes, and the secreted factors may induce the recruitment of cells from the surrounding tissues and promote regeneration of injured tissue [15]. To this regard, the SDF-1/CXCR4 axis has been shown to play a key role in endogenous and transplanted stem cell homing in the injured site promoting the regeneration of different tissues including cartilage [16, 17].

Another key player in cell adhesion is integrin *α*4*β*1 (very late antigen-4, VLA-4). It has been demonstrated a crucial link between the CXCR4/SDF-1 homing axis and the VLA-4/VCAM-1 (vascular cell adhesion molecule-1, CD106) adhesion axis [18]. In particular, SDF-1 (through CXCR4) increases VLA-4 adhesion to VCAM-1. VLA-4 is an integrin dimer composed of *α*4 (CD49d) and *β*1 (CD29) [19]. Although MSCs lack the expression of selectins, they express integrin *β*1. Interestingly, in contrast to BM-MSCs, UC-MSCs express integrin *α*4, VCAM-1, and intercellular adhesion molecule-1 (ICAM-1; CD54) supporting their stronger potential in homing [20].

One more ligand of integrin *β*1 is osteopontin (OPN), an osteogenic marker with several biological functions including migration, adhesion, and survival of MSCs [21]. On the other hand, OPN is also involved in regulation and propagation of inflammatory responses of macrophages, T-cells, and dendritic cells [22]. Notably, OPN is involved in different inflammatory pathologies including RA and OA pathogenesis [23, 24]. In a study of Schneider et al., UC-MSCs showed similar osteogenic and migration abilities compared to BM-MSCs with the lesser expression of OPN and the major expression of matrix metalloproteinases (MMP)-1 and -2 [25].

Moreover, extensive evidence found that growth factors play an important role in homing and migration of MSCs, as seen for basic fibroblast growth factor (bFGF), VEGF, HGF, IGF-1, PDGF, and transforming growth factor *β*1 (TGF-*β*1) [26]. In particular, UC-MSCs are able to migrate *in vitro* and *in vivo*, in response to chemotactic factors such as EGF, FGF-2, HGF, IGF-1, PDGF-BB, TGF-*β*, and VEGF, along with SDF-1, MCP-1, and VCAM-1 [15].

### 2.2. Capacity of Adaptation to Cartilage Hypoxic Environment

Because of the lack of vascularization, the physiological oxygen tension (physioxia) within human articular cartilage ranges between 2 and 5% [27]. Therefore, any MSC candidate for stem cell therapy of cartilage diseases should be able to adapt to a hypoxic environment with limited nutrient supply while maintaining its regenerative properties. Oxygen tension ranges from 1%-7% in bone marrow and from 10%-15% in adipose tissue [28, 29]. Regarding perinatal tissues such as the UC, oxygen tension within the mammalian female reproductive tract is low, between 1.5% and 8%, and lasts throughout the fetal development with dissolved oxygen in the fetal circulation rarely exceeding 5% [30]. Moreover, the UC is supplied by only two arteries and one vein and lacking in capillaries or lymphatics suggesting that UC-MSCs are physiologically adapted to survive in a hypoxic environment. It has been shown that low oxygen tension increases UC-MSC proliferation potential and matrix production and enhanced chondrogenic marker expression in UC-MSCs [31, 32]. This increased chondrogenic differentiation can lead to hypoxia-inducible factor-1 alpha (HIF-1*α*) and HIF-2*α* increased expression, NOTCH signaling activation, and the subsequent Sox-9 induction [33]. In addition, the UC-MSCs cultured under hypoxic conditions showed increased expression of energy metabolism-associated genes including GLUT-1, LDH, and PDK1 suggesting a switching of cell metabolism from oxidative phosphorylation to anaerobic glycolysis [32]. The yield of lactate production from glucose, however, is significantly lower in UC-MSCs than it has been reported in BM- and AT-MSCs in both hypoxic and normoxic conditions [32]. This finding could be explained by our recent study [34]. We demonstrated that all the three UC-MSC populations (PV-, WJ-, and CL-MSCs) exhibit low levels of mitochondrial and glycolytic activities. Moreover, PV-, WJ-, and CL-MSCs showed comparable mitochondrial respiration parameters in both normal and oxygen and glucose deprivation followed by reperfusion (OGD/R) conditions maintaining their proliferation capacity. Interestingly, PV-MSCs showed the highest oxygen consumption rate and OGD/R affected their metabolism but not their viability suggesting a superior mitochondrial activity compared to the other UC-MSC populations. While CL-MSCs were the cells least affected suggesting their robust survival in ischemic environment. These evidences taken together suggest that UC-MSCs may be a pivotal source for stem cell-based therapy of ischemic pathologies including chondropathies, brain, heart, and lung diseases [35–38]. Further investigations are needed to better understand whether these slight but significant differences among the three UC-MSCs are due to the specific region's composition of different number of healthy mitochondria or improved adaptation of mitochondria to ischemic conditions.

### 2.3. Promotion of Survival, Proliferation, and Differentiation

MSCs secrete growth factors that are involved in several biological processes such as homing and migration as well as promotion of survival, proliferation, and differentiation. Some of growth factors with a key role in cartilage repair are bone morphogenetic proteins (BMPs), epidermal growth factor (EGF), HGF, IGF, PDGF, VEGF, FGF, and TGF families and UC-MSCs are a rich source of them [39].

EGF is one of the ligands of EGF receptor (EGFR) that plays a key role in joint homeostasis. In particular, EGFR stimulates chondrocyte proliferation and survival as well as maintenance of cartilage in adulthood. On one hand, EGFR signaling promotes the lubrication of the articular surface by increasing the boundary lubricants Prg4 and HA from superficial chondrocytes [40]. On the other hand, EGFR signaling also can play a catabolic action by inhibiting the expression of the chondrogenic master transcription factor Sox9, thereby suppressing the synthesis of cartilage matrix proteins, such as type II collagen (Col II) and aggrecan, as well as by stimulating the expression of MMPs involved in cartilage degradation, such as MMP-13 [41]. Interestingly, Zhang et al. recently showed the UC-MSCs release EGFR ligands TGF-*α* and EGF attenuating OA progression via EGFR signaling pathway of cartilage superficial layer cells [42]. In addition, UC-MSCs inhibited the apoptosis of chondrocytes, increased the expression of chondrogenesis-related genes (Col-2, Sox9), and reduced the expression of cartilage catabolism-related genes (MMP-13, ADAMTS-5) *in vitro* and *in vivo* [42].

HGF is a multifunctional growth factor that affects cell survival and proliferation, matrix metabolism, inflammatory response, and neurotrophic action playing an important role in normal bone and cartilage turnover [43]. In particular, HGF and VEGF can reduce tissue injury, inhibit fibrotic remodeling and apoptosis, promote angiogenesis, stimulate stem cell recruitment and proliferation, and reduce oxidative stress [44]. A recent comparative study showed that the secretion of HGF was three times higher in UC-MSCs compared to AT-MSCs and around nine times higher than in BM-MSCs [45]. In contrast, UC-MSCs secreted the lower levels of VEGF-A. This is probably due to the fact that VEGF-A and HGF signaling pathways reciprocally modulate each other [46].

IGF1 has been implicated in promotion of chondrogenesis and accumulation of cartilage-specific ECM molecules [47]. In addition, the synergy between TGF-*β*3 and IGF-1 promotes intervertebral disc regeneration [48]. WJ contains large amounts of IGF-I and IGF-I-binding proteins BP-3 and BP-1 suggesting a key role in stimulation of UC-MSCs to produce collagen and glycosaminoglycans (GAGs) in UC matrix as well as influencing the chondrogenic differentiation of these cells [49, 50].

TGF-*β* superfamily consists of about 30–35 different proteins including TGF-*β* proteins (TGF-*β*1-*β*2-*β*3), Bone Morphogenetic Proteins (BMPs), and Growth Differentiation Factors (GDFs) involved in chondrogenic differentiation and production of cartilage extracellular matrix as well as stimulation of cartilage repair [51]. TGF-*β*1, -*β*2, and -*β*3 play key roles in regulation of chondrocyte differentiation from early to terminal stages, including condensation, proliferation, terminal differentiation, and ECM synthesis as well as maintenance of articular chondrocytes [52]. All the three isoforms are expressed in mesenchymal condensations and secreted by UC-MSCs [53–55]. BMPs play important roles in bone and cartilage formation, including various aspects of embryonic development, such as skeletogenesis, and hematopoietic and epithelial cell differentiation [56]. Moreover, BMPs can induce protection against cartilage damage caused by inflammation or trauma, as well as stimulation of regenerative processes. BMPs are classified into subfamilies, including BMP subfamily (from BMP1 to BMP15), the osteogenic protein (OP) subfamily (OP1, OP2, and OP3 also known as BMP7, BMP8, and BMP8b, respectively), the GDF subfamily (GDF1, GDF2/BMP9, GDF3, GDF5/BMP14, GDF6/BMP13, GDF7/BMP12, GDF8, GDF9, GDF10, and GDF11/BMP11), and the cartilage-derived morphogenetic proteins (CDMP1/BMP14 and CDMP2/BMP13) [56]. BMP2, 4, 6, 7, and 9 have been reported to induce *in vitro* chondrogenesis of human MSCs [57]. UC-MSCs have been demonstrated to secrete BMP2 *in vitro* and to induce the increase of endogenous BMP4, 5, and 7 levels *in vivo* [58–60]. In addition, UC-MSCs induce overexpression of GDF5/BMP14/CDMP1, promoting chondrogenic differentiation in cocultures with fibroblast-like synoviocytes, thus, suggesting their potential in cartilage repair [61]. Moreover, UC-MSCs respond to BMP6 via decapentaplegic homolog (SMAD) signaling (SMAD 1/4/5, BMPR1A, and BMPR2 receptors) enhancing osteogenic differentiation [62]. In particular, BMP-2 stimulates osteogenesis as well as matrix synthesis, promoting cartilage repair (by upregulation of tissue inhibitors of metalloproteinases-1, TIMP-1) and reversing chondrocyte dedifferentiation [63]. BMP-7 promotes cartilage matrix synthesis by acting synergistically with other anabolic growth factors and also inhibits catabolic factors, such as matrix metalloproteinase-1 (MMP-1), MMP-13, IL-1, Il-6, and IL-8 [64].

### 2.4. Cartilage Extracellular Matrix Repair

UC-MSCs can increase the ECM synthesis and inhibit the cartilage ECM destruction supporting the tissue repair. UC stromal tissue shares a number of features with cartilage ECM: UC-MSCs are able to synthetize aggrecan, type II collagen, and express SOX-9 transcription factor [12]. The deposition of ECM molecules and regulation of MMPs and their inhibitors (TIMPs) are the main mechanisms involved in cartilage ECM synthesis. MSCs secrete high levels of TIMP-1 and TIMP-2, which inhibit MMP-9 and MMP-2, respectively, thus, suppressing cartilage ECM resorption [65]. UC-MSCs secrete MMP-2, -8, -9, and -13 as well as TIMP-1 and TIMP-2 suggesting a balance between protection of ECM and antifibrotic activity (Table 2) [66–68]. In addition, UC-MSCs secrete growth factors such as HGF, IGF-1, and TGF-*β* superfamily members that stimulate cartilage ECM synthesis. In particular, HGF has been involved in inhibition of the fibrosis and apoptosis of chondrocytes and increase ECM synthesis [65]. IGF-I and IGF-I-BP-3 and -BP-1 stimulate UC-MSCs to produce collagen and glycosaminoglycans (GAGs) [49]. BMP-2 increases TIMP-1 expression while BMP-7 inhibits MMP-1 and MMP-13 suppressing the ECM degradation [63, 64].

### 2.5. Anti-Inflammatory and Immunomodulatory Properties

The microenvironment of damaged articular cartilage is particularly challenging, due to hypoxia, insufficient blood supply, and concurrent inflammation. The latter contributes to the degeneration of the joints because it hampers the proliferation of chondrocytes and the deposition of cartilage matrix, resulting in low efficiency of repair. Immunomodulatory and anti-inflammatory properties of UC-MSCs have been widely described (Table 2) [69]. In particular, UC-MSCs express MHC class I (HLA-ABC) at low levels and lack MHC class II (HLA-DR, -DP, and -DQ). Moreover, they express other molecules belonging to noncanonical type I MHC such as HLA-G, HLA-E, and HLA-F [70–72]. Interestingly, HLA-G interacts with Ig-like transcript (ILT) receptors (ILT-2, ILT-3, and ILT-4), which are expressed by T and B lymphocytes, as well as natural killer (NK) cells and mononuclear phagocytes [69]. Through this interaction, HLA-G displays relevant immune functions which physiologically contribute to maternal-fetal immunotolerance. In addition, UC-MSCs lack CD40/CD40L, CD80, CD86, and B7 costimulatory antigens implicated in the activation of T and B cell responses and express coinhibitory molecules including B7-H3/CD276, CD73, Indolamine 2,3-dioxygenase-1 (IDO-1), Galectin-1 (Gal-1), and leukemia inhibitory factor (LIF) [73]. WJ-MSCs showed an immunosuppressive function by inhibiting the proliferative response of T helper cells (Th/CD4+) Type 1 (Th1) and Th17 and increasing Th2 and regulatory T cells (Tregs) [74]. UC-MSCs have been shown to be able to suppress the proliferation of both CD4 and CD8 cytotoxic T lymphocytes (Tc) and to decrease proinflammatory IFN-*γ* in activated peripheral blood mononuclear cells (PBMCs) [54, 75]. Moreover, secreted factors such as HGF and TGF-*β*1 may function as mediators for T cell suppression [76, 77]. UC-MSCs are also able to inhibit B-cells and natural killer (NK) cell proliferation as well as regulate monocyte/macrophage system by reducing the infiltration of macrophages in injured tissues and shifting macrophages toward a M2 anti-inflammatory phenotype [78, 79].

In the synovia of OA patients, various immune cells have been identified including M1 macrophages, T cells Th1, Th17 and Tc, and B cells, leading to chronic inflammation, exacerbation of arthritis, and tissue damage [80]. UC-MSCs have been shown to reduce synovial inflammatory cells infiltration, such as CD4+ T cells and macrophages, as well as significantly decrease the expression of interleukin- (IL-) 1*β* and tumor necrosis factor-*α* (TNF-*α*), while increasing anti-inflammatory factors TNF-*α*-induced protein 6 (TSG-6) and IL-1 receptor antagonist (IL-1RA) in rat OA models induced by monosodium iodoacetate (MIA) [81, 82]. In another study of MIA-induced OA in rabbits, UC-MSCs showed a prominent cartilage protective effect due to upregulation of growth factors FGF-2, TGF-*β*1, and IGF-1, secretion of ECM molecules (collagen type-I alpha-1 chain, collagen type-II alpha-1 chain, and aggrecan), reduction of the expression levels of proinflammatory cytokines Tnf-*α*, IL-1*β*, IL-6, and IL-17, and increase of anti-inflammatory cytokines TGF-*β*1, IL-10, and IL-1RA [83]. Interestingly, our group and others showed that UC-MSCs keep their hypoimmunogenic and immunomodulatory properties even when they had undergone *in vitro* chondrocyte differentiation [50, 84].

RA is a chronic inflammatory autoimmune disease characterized by chronic proliferation of synovial cells and progressive joint damage [85]. Fibroblast-like synoviocytes (FLS) play an important role in thickening of the synovium determining arthritis and cartilage degradation as well as inflammation and degradation of the joints. UC-MSCs inhibit Cadherin 11 expression in RA FLS by secreting IL-10. This event precludes the ability of FLS from RA patients to migrate and erode cartilage of other joints, thereby improving arthritis [86].

## 3. Preclinical and Clinical Studies of UC-MSCs for the Treatment of Cartilaginous Diseases

Thanks to their chondrogenic potential and immunomodulatory and anti-inflammatory properties, as well as their ability to promote endogenous repair mechanisms, UC-MSCs have been regarded as potential therapeutic agents against cartilage degradation. In particular, early evidences that emerged from *in vitro* studies on cell cultures (summarized in Table 3) have been confirmed in several *in vivo* animal models (listed in Table 4) and in recent clinical trials (reported in Table 5).

Preliminary *in vitro* studies investigated the chondrogenic potential of UC-MSCs, demonstrating their ability to achieve both hyaline, fibrous, and elastic cartilage phenotypes as well as nucleus pulposus-like cell differentiation capacity [87–90]. In addition, also the osteogenic, adipogenic, and myogenic differentiation potential have been reported suggesting UC-MSCs could be a pivotal stem cells source for tissue engineering applications in orthopaedics [91]. Comparative studies reported slightly differences in chondrogenesis between the UC-, BM-, and AT-MSCs. In particular, according to Danišovič et al., BM-MSCs showed the best chondrogenic potential while Hildner and coworkers showed that differentiated UC-MSCs present a more fibrous than hyaline cartilage phenotype compared to AT-MSCs suggesting their role in regeneration of fibrocartilage-like meniscus [87, 92]. Moreover, the results of Wu and coworkers indicate that, although nucleus pulposus stem/progenitor cells (D-NP-MSCs) isolated from degenerated intervertebral disc (IVD) shared the MSCs characteristics with UC-MSCs, the latter showed better proliferation capacity and differentiation potential, suggesting that UC-MSCs as a suitable source for regenerative therapy of IVD degeneration [93]. Furthermore, UC-MSCs may be an attractive alternative to condylar cartilage cells for temporomandibular joint tissue engineering applications [94]. Interestingly, UC-MSCs displayed superior anti-inflammatory, immunomodulatory, and trophic effects compared to adult MSCs including articular cartilage (AC), Hoffa's fat pad (HFP), synovial membrane (SM), and maintain their immunomodulatory and anti-inflammatory properties after differentiation [50, 84, 95]. Moreover, coculture experiments of UC-MSCs and articular cartilage cells (ACs), fibroblast-like synoviocytes (FLSs), and nucleus pulposus cells (NPCs) showed their suitability for the treatment of arthritis, synovitis, and IVD degeneration [61, 96–98]. Finally, there are several cartilage tissue engineering studies that demonstrated the osteochondral differentiation capacity of UC-MSCs in different scaffold constituted by acellular cartilage extracellular matrix (ACECM), alginate enriched in hyaluronic acid (Alg/HA), polycaprolactone/collagen (PCL/Coll), polyglycolic acid (PGA), poly L-lactide/D-lactide/glycolide (PLGA), poly-L-lactic acid (PLLA), polyvinyl alcohol-polycaprolactone (PVA-PCL), and silk fibroin/hyaluronic acid (SF/HA).

*In vivo* studies in different animal models from mice to horses confirmed *in vitro* studies showing the feasibility of using UC-MSCs for the treatment of IVD degeneration, OA, RA, and cartilage defects repair. UC-MSC transplantation promotes chondrogenesis and improves the histology, cellularity, and ECM proteins content along with reduction of inflammation in a preclinical model of IVD degeneration. In the same way, UC-MSCs induce regeneration and repair of cartilage reducing its destruction, promote recovery from movement impairment, and reduce joint effusion and inflammation slowing down the progression of OA animal models. In addition, preclinical studies on RA treatment showed that UC-MSCs exerted the best therapeutic effect in reducing bone resorption, joint destruction, and inflammatory factor expression compared to BM-MSCs. Interestingly, several evidences support the regenerative potential of UC-MSCs in cartilage and osteochondral defects repair.

Following the promising *in vitro* and *in vivo* results, clinical applications have been attempted using UC-MSCs for the treatment of OA and RA (Table 5). In summary, clinical trials for the treatment of OA showed significant amelioration of pain and disability at 6 and 12 months of follow-up. No severe adverse events were reported. The main outcomes for RA patients treated with UC-MSCs were significant reduction of RA serological markers and improvement of health index and joint function index 1 year after treatment. No new or unexpected safety issues in 1-year follow-up. Despite the promising results of clinical trials, further basic and translational research investigations are needed to better understand the best stem cell candidate, scaffold materials, and/or best cellular derivatives which can be suitable for the different types of cartilage regeneration. In parallel, there is the need to increase the knowledge about underlying regenerative mechanisms. Finally, more research is needed to convert preclinical evidences obtained in animal models, to human-based clinical applications for cartilage regeneration. Consensus is still lacking in key points such as the methods to obtain the cell source, the use of scaffolds as well as bioactive molecules in parallel to the administration of stromal cells. As shown in the human studies reviewed so far, the achievement of amelioration of some parameters and confirmation of the safety of the overall procedure still needs more data generated on the interaction of the transplanted cells with the host tissue, their proper differentiation *in vivo*, as well as the long-term achievements of this cellular replacement strategy.

## 4. Conclusions

In conclusion, UC-MSCs represent a promising candidate for the therapy of chondropathies, as highlighted by the encouraging results emerged from *in vitro* and *in vivo* investigations and from the available results from clinical trials. UC-MSCs are characterized by several potential advantages such as a frank multilineage differentiation potential, immunomodulatory, and anti-inflammatory properties, as well as MSCs the ability to constitutively produce molecules that are involved in cartilage matrix biogenesis and in the trophic and reparative functions. In addition, UC-MSCs are able to migrate, home, and survive in an ischemic and nutrient-poor environment like cartilage as well as to produce an extracellular matrix (ECM) similar to that and induce endogenous repair mechanisms. We believe that these results warrant the need for further researches that can better define the criteria leading to the adoption of UC-MSCs in the stem cell-based therapy of cartilage diseases, as well as characterizing the mechanism of repair and increase the knowledge on the biomechanical properties of the regenerated cartilage tissue in vivo.

## Figures and Tables

**Table 1:** Comparative analysis of the advantages and disadvantages of different stem cell populations used for cartilage regeneration.

Source	Advantages	Disadvantages	Ref.
ESCs	Pluripotent Chondrocytes differentiation capacity Synthesis of cartilage ECM	Difficulty of controlling ESCs differentiation Ethical concerns Risk of immune rejection Risk of teratoma formation	[99]
iPSCs	Pluripotent Chondrocytes differentiation capacity Synthesis of cartilage ECM No ethical concerns	Complex and expensive iPSC generation procedures Risk of immune rejection Risk of teratoma formation	[100]
BM-MSCS	Multipotent Chondrocytes differentiation capacity Synthesis of cartilage ECM No ethical concerns Low immunogenicity	Invasive isolation procedure with risk of infection Low isolation yield (about 1 in 1 × 10^5^ cells in the BM) Donor age affects initial yield of isolation and the proliferative and differentiation properties Sign of senescence from passage 4	[101]
AT-MSCs	Multipotent Chondrocytes differentiation capacity Synthesis of cartilage ECM No ethical concerns Low immunogenicity Isolation yield 500 times more than BM-MSCs	Invasive isolation procedure with risk of infection Heterogeneity of AT-MSCs extracted from different body sites Sign of senescence from passage 8	[101]
UC-MSCs	Pluripotent without teratoma formation risk Chondrocytes differentiation capacity Synthesis of cartilage ECM No ethical concerns Low immunogenicity No risk for the donor High isolation yield (about 1 × 10^4^ cells from 1 cm of cord) No sign of senescence or abnormality over 16 passages 300 fold expansions reached within 6-7 passages	Limited knowledge about the UC-MSCs populations	[102]
IPFSCs	Multipotent High chondrogenic potential	Limited source of tissue (7.5 million cells from 5 g of tissue)	[103]
SD-MSCs	Multipotent Higher chondrogenic potential than BM-MSCs Higher colony-forming potential and proliferation rate than BM- and AT-MSCs	Limited source of tissue (knee: 10.5 ± 8.1 × 10^3^ cells/mg; hip: 3.1 ± 2.2 × 10^3^ cells/mg)	[104]

AT-MSCs: adipose tissue-derived mesenchymal stem cells (MSCs); BM-MSCs: bone marrow-derived MSCs; ESCs: embryonic stem cells; IPFSCs: infrapatellar fat pad-derived stem cells; iPSCs: induced pluripotent stem cells; UC-MSCs: umbilical cord-derived MSCs; SD-MSCs: synovium-derived MSCs.

**Table 2:** Expression analysis of different markers involved in homing and migration.

Marker	UC-MSCs	BM-MSCs	AT-MSCs	Ref.
Secreted factors				
COX-2	*↑*	↓	↓	[45]
bFGF (FGF-2)	*↑↑*	↓	*↑*	[45]
HGF	*↑↑*	↓	*↓↓*	[45, 105]
IGF-1	*↑*	↓	↓	[54]
IL-1*α*	*↑↑*	↓	ND	[54]
IL-1𝛽	*↑↑*	↓	*↑*	[54, 105, 106]
IL-6	*↑*	↓	↓	[105]
	*↔*	*↔*	*↔*	[45]
IL-8	*↑*	↓	↓	[105]
IL-1RA	*↑*	↓	↓	[105]
MCP-1 (CCL2)	*↑*	↓	↓	[45, 105]
	*↔*	*↔*	*↔*	[45]
MIP-1*α* (CCL3)	↓	*↑*	*+*	[107, 108]
MIP-1𝛽 (CCL4)	*+*	*-*	*-*	[105]
OPN	↓	*↑*	↓	[109]
PDGF-AA	*↑*	↓	↓	[105, 107]
PDGF-AB	*↑*	↓	*-*	[107, 110]
PDGF-BB	*-*	↓	*-*	[105]
PGE2	↓	↓	↓	[106]
PlGF	↓	*↓↓*	*↑*	[105]
SDF-1	*↑*	*↑*	↓	[111, 112]
SDF-1*α*	*↔*	*↔*	*↔*	[45]
TGF-𝛽1	*↔*	*↔*	*↔*	[105]
TGF-𝛽2	*↑*	↓	↓	[105]
VEGF-A	*↑*	↓	↓	[45]
VEGF-D	*↑*	↓	↓	[105, 107]
MMPs and TIMPS				
MMP-1	*↑*	↓	*↑↑*	[105]
MMP-2	*↔*	*↑*	*↔*	[113]
MMP-3	↓	↓	*↑*	[105]
MMP-7	↓	*↑*	*-*	[105]
MMP-8	*-*	*↑*	*-*	[105]
MMP-13	*-*	*-*	*+*	[105]
TIMP-1	*↔*	*↔*	*↔*	[113]
TIMP-2	*↔*	*↔*	*↔*	[113]
Adhesion molecules and receptors				
CCR2	*↑*	↓	↓	[54]
CXCR4	*↑*	↓	↓	[114]
Flt-1 (VEGFR1)	*↑*	↓	ND	[54]
ICAM-1	*↑↑*	↓	*↑*	[45]
Integrin *α*4	*↑↑*	↓	*-*	[54]
PDGF-Ra	*↔*	*↔*		[107]
PDGF-Rb	*↑*	↓	↓	[107]
VCAM-1	↓	*↑*	↓	[45]
Immunoregulatory molecules				
B7-H3 (CD276)	*+*	*+*	*+*	[71]
CD200	*↑*	↓	*↓↓*	[54, 115]
Galectin 1	*↑*	*↑*	↓	[113]
HLA-ABC	*↔*	*↔*	*↔*	[54, 116]
HLA-DR	*↔*	*↔*	*↔*	[54, 116]
HLA-G	*↑*	↓	↓	[54, 116]
HLA-E	*↑*	↓	↓	[54, 117]
HLA-F	*↑*	↓	*-*	[54, 117]
IDO	*↔*	*↔*	*↔*	[45]
	*↑↑*	*↑*	ND	[118]
IP-10	*↑*	↓	↓	[105]
LIF	*↑*	↓	↓	[54, 113]
PD-L1 (B7-H1)	*↑*	↓	*+*	[54, 119]
PD-L2 (B7-DC/CD273)	*↑*	↓	*+*	[54, 119]
RANTES (CCL5)	*↑*	↓	↓	[105]
TLR-1	*↔*	*↔*	*↔*	[106]
TLR-2	*↔*	*↔*	*↔*	[106]
TLR-3	*↔*	*↔*	*↔*	[106]
TLR-4	*-*	*↔*	*↔*	[106]
TLR-5	*↔*	*↔*	*↔*	[106]
TLR-6	*↔*	*↔*	*↔*	[106]
TLR-9	*↔*	*↔*	*↔*	[106]

Expression: ↑: higher; ↑↑: significantly higher; ↓: lower; ↓↓: significantly lower; ↔: similar; +: qualitatively expressed, but not quantified; -: not expressed; ND: not detected.

**Table 3:** In vitro studies of UC-MSCs or their secretome.

Study type	Source	Aim	Culture system	Results	Ref.
Chondrogenic differentiation	Human UC-MSCs	UC- and AT-MSC comparison	Cultured in CM supplemented with TGF*β*3 and BMP-6	A more fibrous than hyaline cartilage phenotype in UC-MSCs compared to AT-MSCs	Hildner et al., 2010 [87]
	Human WJ-MSCs	Differentiation into NP-like cells	Coculture with NPCs	Increased expression of aggrecan, collagen II, and SRY-type HMG box-9 genes	Ruan et al., 2012 [88]
	Human UC-MSCs	Differentiation into NP-like cells	Cultured in a laminin-rich pseudo-3D culture system	GAGs, collagen II, laminin *α*5, and laminin receptors (integrin *α*3 and *β*4) expression	Chon et al., 2013 [89]
	Human WJ-MSCs	Immunomodulatory properties test	Cultured in CM	Differentiated WJ-MSCs maintain their immune privilege	La Rocca et al., 2013 [50]
	Human UC-MSCs	Elastic cartilage differentiation	Seeded on PLGA nanofiber scaffolds with CM and CTGF	Increase of GAG/DNA ratio, collagen II, elastin mRNA and protein. No difference in collagen X or fibrillin mRNA	Caballero et al., 2013 [90]
	Human UC-MSCs	Tissue-engineered (TE) elastic cartilage from UC-MSCs and human cartilage comparison	Seeded onto PLGA nanofiber scaffolds with CM supplemented with CTGF	TE elastic cartilage from UC-MSCs expresses embryonic fibrillin III and similar levels of elastin, fibrillin I, collagens I and X when compared to native cartilage.	Pappa et al., 2014 [120]
	Human and porcine UC-MSCs	Effects of periodic vibratory stimulus on UC-MSC differentiation	Cultured in chondrogenic or osteogenic medium and exposed to 1 or 100 Hz frequency vibrations	1 Hz stimulation resulted in a cartilage phenotype while 100 Hz stimulation resulted in a bone phenotype for both human and porcine UC-MSCs	Cashion et al. 2014 [121]
	Human UC-MSCs	UC-, BM-, and AT-MSC chondrogenesis comparison	Cultured in CM	Slightly differences in chondrogenesis between the MSCs. BM-MSCs showed the best chondrogenic potential	Danišovič et al., 2016 [92]
	Human UC-MSCs	Effect of mechanical compression on UC-MSC chondrogenesis	Seeded in PVA-PCL scaffold with CM and subjected to dynamic compression	Increase in chondrogenic differentiation	Remya et al., 2016 [122]
	Human WJ-MSCs	Simulation of the articular cartilage microenvironment	Coculture of WJ-MSCs and primary ACs in ACECM- oriented scaffold	Chondrogenic differentiation of WJ-MSCs without any inducer, hyaline cartilage phenotype, and improved cytoactivity of ACs	Zhang et al., 2019a [96]
	Human UC-MSCs	Interactions between ACs and UC-MSCs.	Coculture with direct cell-cell contact	Enhanced differentiation of UC-MSCs and reduced dedifferentiation of chondrocytes	Li et al., 2019 [97]
	WJ-MSCs	Immunomodulatory properties test	Chondrogenic differentiation in Alg/HA scaffold	Differentiated WJ-MSCs inhibit T cell alloproliferation and maintain paracrine activity and functional immunomodulation	Voisin et al., 2020 [84]
Cartilage tissue engineering	Human UC-MSCs	PGA and PLLA scaffolds comparison	Seeded on nonwoven PGA or PLLA scaffolds in CM	Similar chondrogenic potential of UC-MSCs in PLLA and PGA scaffolds.	Zhao et al., 2010 [123]
	Human WJ-MSCs	WJ- and BM-MSCs chondrogenesis comparison	Seeded in PCL/Coll nanofibrous scaffolds in CM	Enhanced cell attachment, proliferation, and chondrogenesis of WJ-MSCs over BM-MSCs	Fong et al., 2012 [124]
	Human UC-MSCs	Chondrogenic differentiation	Embedded in collagen hydrogel scaffold with CM	Increased expressions of collagen II, aggrecan, COMP, and sox9	Chen et al., 2013 [125]
	Human UC-MSCs	Chondrogenic differentiation in PVA-PCL scaffolds	Seeded in PVA-PCL scaffolds with individual TGF*β*1, TGF*β*3, IGF, BMP2 and their combination with BMP2	SOX9, collagen II and aggrecan expression. The combination TGF-*β*3 and BMP-2 was the more effective for chondrogenesis	Nirmal et al., 2013 [126]
	Human WJ-MSCs	Fabrication of a nonscaffold tissue-engineered cartilage	Pellet culture combined with RCCS	RCCS formed larger and condenser cartilage-like tissue enriched of GAGs and collagen II than pellet culture	Liu et al., 2014 [12]
	Human WJ-MSCs	WJ- and BM-MSCs chondrogenesis in agarose hydrogel	Encapsulation of WJ-MSCs or BM-MSCs aggregates in agarose hydrogels	Both BM-MSCs and WJ-MSCs did better in matrix biosynthesis and chondrogenesis when in aggregates than in free cell suspension	Sridharan et al., 2015 [127]
	Human UC-MSCs	Chondrogenic differentiation in SF/HA scaffold	Seeded in different ratios of SF/HA with CM	Expression of collagen II, aggrecan, and Sox9. SF80 and SF70 scaffolds are the best for chondrogenesis	Jaipaew et al., 2016 [128]
	Human WJ-MSCs	Chondrogenesis of WJ-MSCs in PLLA-collagen nanofibers scaffold	Seeded on PLLA-collagen nanofibers scaffold with CM	PLLA-collagen nanofibers scaffold promotes the chondrogenic differentiation of WJ-MSCs	Wang et al., 2017 [129]
	Human WJ-MSCs	Chondrogenesis of WJ-MSCs in hyaluronic acid-based hydrogels	Seeded in hyaluronic acid-based hydrogels with CM	Increase of GAGs, collagen II and aggrecan,	Aleksander-Konert et al., 2016 [130]
	Human UC-MSC- ECM	Effect of decellularized UC-MSC-ECM on ACs	ACs seeded in culture plates coated with UC-MSC-ECM	Promotion of the proliferation and differentiation of chondrocytes	Zhang et al., 2019b [131]
Fibrocartilage tissue engineering	Human UC-MSCs	UC- and BM-MSCs chondrogenesis comparison	Seeded onto PGA scaffolds in chondrogenic medium	More GAGs, collagen I, and aggrecan and less collagen II in UC-MSCs than BM-MSCs	Wang et al., 2009a [132]
	Human UC-MSCs	Best density for UC-MSCs chondrogenesis	Seeded on nonwoven PGA scaffold in CM	More collagen I and II, aggrecan, GAGs, and mechanical integrity in high-density groups	Wang et al., 2009b [133]
Osteochondral tissue engineering	Human UC-MSCs	Chondrogenic and osteogenic differentiation	Seeded between chondrogenic and osteogenic PLLA constructs	Both chondrogenic and osteogenic differentiation of UC-MSCs in the respective sides of constructs	Wang et al., 2011 [134]
	Human UC-MSCs	Chondrogenic and osteogenic differentiation	Seeded in osteogenic scaffold and in Collagen I and III- or HA-based chondrogenic scaffolds in normoxic or hypoxic (8% O2) conditions.	Both chondrogenic and osteogenic differentiation of UC-MSCs. Hypoxia improved the expression of these chondrogenic markers	Marmotti et al., 2017 [31]
Orthopaedic tissue engineering	Human UC-MSCs	Multilineage differentiation	Cultured in adipogenic, osteogenic, chondrogenic, or myogenic medium	Multilineage differentiation potential toward bone, fat, cartilage, and muscle	Marmotti et al., 2012 [91]
IVD degeneration	Human UC-MSCs	UC- and D-NP-MSCs comparison	Cultured with CM	D-NPMSCs expressed lower expression levels of CD29 and CD105, reduced proliferation capability and differentiation potentials	Wu et al., 2017 [93]
	Human WJ-MSCs	Interactions between WJ-MSCs and degenerative NPCs	Coculture with or without direct cell-cell contact	NP-like cell differentiation of WJ-MSCs and biological status of degenerative NPCs restoration. The direct cell-cell contact yielded more favorable gene expressions	Han et al., 2018 [98]
	Human UC-MSCs secretome	UC-MSC-conditioned medium (CM) effect on damaged NP-MSCs	Treatment of high glucose-induced degradation of NP-MSCs with UC-MSCs-CM	Reduction of apoptosis and ECM degradation via the p38 MAPK pathway	Qi 2019 et al., 2019 [135]
	Human UC-MSCs-ECM	Effect of UC-MCS-ECM on IVD cells	IVD cells seeded on decellularized UC-MSCs-ECM	UC-MSCs-ECM improved the degenerated phenotype of human IVD cells affecting the expression of Sox2, Sox 9 and TRPS1	Penolazzi et al., 2020 [136]
OA	Human UC-MSCs secretome	Comparison of articular cartilage (AC), Hoffa's fat pad (HFP), synovial membrane (SM), and UC-MSC secretomes	Secretome analysis by mass spectrometry and effect on AC chondrogenesis and immunosuppressive and anti-inflammatory effects on PBMCs and macrophages	UC-MSCs-CM displayed superior anti-inflammatory, immunomodulatory and trophic effects compared to adult MSCs	Islam et al., 2019 [95]
RA	Human UC-MSCs	UC-MSCs effect on FLS	Coculture	Increase of FLS apoptosis, collagen II, and aggrecan; decrease of IL-1*β*, IL-6 and CCL-2	Zeng et al., 2016 [61]
TMJ disorders	Human UC-MSCs	UC-MSCs and TMJ condylar chondrocytes comparison	Seeded in PGA scaffolds in CM	More collagen I and II, GAGs, and cellular density in UC-MSCs than TMJ construct	Bailey et al., 2007 [94]

AC: articular cartilage cells; ACECM: acellular cartilage extracellular matrix; Alg/HA: alginate enriched in hyaluronic acid; CTGF: connective tissue growth factor; CM: chondrogenic medium; D-NP-MSCs: NP stem/progenitor cells isolated from degenerated IVD; ECM: extracellular matrix; FLS: fibroblast-like synoviocytes; GAGs: glycosaminoglycans; n.a.: not applicable; IVD: intervertebral disc; NP: nucleus pulpous; NPCs: nucleus pulposus cells; OA: osteoarthritis; PCL/Coll: polycaprolactone/collagen; PGA: polyglycolic acid; PLGA: poly L-lactide/D-lactide/glycolide; PLLA: poly-L-lactic acid; PMEF: pulsed electromagnetic field; PVA-PCL: polyvinyl alcohol-polycaprolactone; RA: rheumatoid arthritis; RCCS: rotary cell-culture system; SF/HA: silk fibroin/hyaluronic acid; TMJ: temporomandibular joint.

**Table 4:** In vivo studies of cartilage repair with UC-MSCs or their secretome.

Pathology	Source	Host	Study design	Results	Ref.
IVD degeneration	Human UC-MSCs	Rabbit	1 × 10^5^ UC-MSCs injected into degenerated IVD	Increase in cellularity and a relative preservation of architecture	Leckie et al., 2013 [137]
	Human UC-MSCs	Rabbit	1 × 10^6^ UC-MSCs or 1 × 10^6^ UC-MSC-derived CPCs injected into degenerated IVD	Improvement in the histology, cellularity, ECM proteins, water, and GAGs contents and higher expression of NP specific markers SOX9, ACAN, COL2, FOXF1, and KRT19 with CPCs	Beeravolu et al., 2018 [138]
	Human UC-MSCs	Rabbit	1 × 10^6^ UC-MSC-derived NPCs injected into degenerated IVD	Improvement in the histology, cellularity, sulfated GAGs, and water contents of the NP. Expression of SOX9, ACAN, COL2, FOXF1, KRT19, PAX6, CA12, and COMP	Perez-Cruet et al., 2019 [139]
	Human UC-MSCs	Rat	1 × 10^6^ UC-MSCs or UC-MSC-derived CPCs injected into degenerated IVD	Expression of chondrogenic markers and downregulation of pain and inflammatory genes. Differentiation of transplanted UC-MSCs and CPCs in functional NPCs. Better survival, homing, and distribution in IVD with CPCs.	Ekram et al., 2021 [140]
OA	Equine UC-MSCs	Rabbit	Early (day 3) or delayed (day 15) intra-articular injection of 3,5.10^6^ UC-MSCs	Early IA injection of UC-MSCs exerted better anti-inflammatory and anticatabolic effects (reduction of MMPs -1, -3, -13, and TNF-a)	Saulnier et al., 2015 [141]
	AM/UC particulate	Rat	Intra-articular injection of 50 or 100 *μ*g/*μ*L AM/UC particulate decellularized	Attenuation of cartilage destruction, significant increase in cartilage thickness and volume, significant decrease in total lesion area with high dose at 4 weeks postinjection	Raines et al., 2017 [142]
	Human UC-MSCs	Mouse	Intra-articular injection of 1 × 10^5^ UC-MSCs	Regeneration and repair of cartilage, recovery from movement impairment, amelioration of cartilage apoptosis via caspase 3 pathway	Chang et al., 2018 [143]
	CanineUC-MSCs	Dog	Intra-articular injection of 1 × 10^6^ UC-MSCs on days 1 and 3	Repair of cartilage and patella, improvement of the healing of the surrounding tissue, reduction of joint effusion and inflammation (reduction of TNF-*α*, IL-6, and IL-7 blood levels)	Zhang et al., 2018 [144]
	Canine UC-MSCs	Dog	Intra-articular injection of 7 × 10^6^ UC-MSCs	Improvement of clinical signs related to OA in treated dogs	Kim et al., 2019 [145]
	Human UC-MSCs	Rabbit	Intra-articular injection of 1 × 10^5^, 5 × 10^5^ or 1 × 10^6^ UC-MSCs	Chondrogenesis induction, upregulation of the expression of growth factors, ECM markers, and anti-inflammatory cytokines, and reduced expression of proinflammatory cytokines. Medium dose exerted the best effects	Kim et al., 2019 [83]
	Human UC-MSCs	Rat	Intra-articular injection of 1 × 10^7^ UC-MSCs overexpressing miR-140-5p	UC-MSCs overexpressing miR-140-5p significantly enhanced articular cartilage self-repairing in comparison to normal UC-MSCs	Geng et al., 2019 [146]
	Human UC-MSCs	Minipig	Intra-articular injection of a UC-MSCs (5 × 10^6^ cells) and HA composite (4%)	Significant gross and histological improvements in hyaline cartilage regeneration	Wu et al., 2019 [147]
	Equine UC-MSCs	Horse	1 or 2 intra-articular injections (at 1-month interval) of 10 × 10^6^ UC-MSCs	improvement of lameness and total clinical score. No apparent clinical benefit of repeated intra-articular administration	Magri et al., 2019 [148]
	Human UC-MSCs	Mouse	Intra-articular injection of 5 × 10^5^ UC-MSCs at 3 or 6 weeks	Significantly reduction of the loss of joint space and no evidence of an inflammatory response	Perry et al., 2020 [149]
	Human UC-MSCs	Rat	Single (day 1) or three (on days 1, 7 and 14) intra-articular injections of 2.5 x 10^5^ UC-MSCs	Amelioration of cartilage erosion, alleviation of inflammatory cells infiltration and hyperplasia of the synovium by repeated injections. Increase number of SFCs on the articular cartilage surface	Tong et al., 2020 [81]
	Human UC-MSCs	Rat	Intra-articular injection of 1 × 10^6^ UC-MSCs in 100 *μ*L HA	Temporary effects that decelerate the progression of cartilage degeneration, but may not inhibit OA progression in the long-term.	Xing et al., 2020 [150]
	Human UC-MSCs	Mouse	Intra-articular injection of low-dose UC-MSCs or UC-MSC-loaded GMs (3 × 10^4^ cells) or high-dose UC-MSCs (3 × 10^5^ cells)	UC-MSC-GMs promoted cartilage regeneration and inhibited macrophage-mediated synovitis better than low-dose and similar to high-dose UC-MSCs	Zhang et al., 2021 [42]
	Human UC-MSCs	Rat	intra-articular injection of 2.5 × 10^5^ UC-MSCs once a week for 3 weeks	UC-MSCs prevent cartilage degradation, restore the proliferation of chondrocytes, and inhibit the inflammatory response	Zhang et al., 2021 [82]
	Human UC-MSCs	Rabbit	Intra-articular injection of UC-MSCs with GO granular lubricant	UC-MSCs loaded with the GO granular lubricant reduce the inflammatory level and improve the level of biochemical environment in the joint	Wang et al., 2021 [151]
RA	Human UC-MSCs	Mouse	Intraperitoneal injection of 1 × 10^6^ UC-MSCs each day for 5 days	Reduction of the severity of RA, reduced levels of proinflammatory cytokines and chemokines (TNF-*α*, IL-6, and MCP-1) and increased levels of the anti-inflammatory cytokine (IL-10), Th1/Th2 type responses shifting and Tregs induction	Liu et al., 2010 [152]
	Human UC-MSCs	Mouse	Intra-articular injection of 1 × 10^6^ UC-MSCs and/or 100 *μ*g/mL TNF-*α* inhibitor	Inhibition of TNF-*α* decreases cartilage destruction by suppressing the immunogenicity of UC-MSCs	Wu et al., 2012 [153]
	Human UC-MSCs	Rat	Tail vein injection of 1 × 10^6^ UC-MSCs	Markedly increased percentage of Tregs and antithrombin levels, decrease of IL-1, IL-17, TNF-*α*, VEGF, and tissue factor levels	Gu et al., 2015 [154]
	Human UC-MSCs	Mouse	Tail vein injection of 1 × 10^6^ UC-MSCs or BM-MSCs or SHED	UC-MSCs exert the best therapeutic effect in reducing bone resorption, joint destruction, and inflammatory factor expression	Zhang et al. 2019 [155]
	Human UC-MSCs	Rat	Intravenous injection of 2 × 10^6^ UC-MSCs	Improvement arthritis, delay of radiological progression, and inhibition of synovial hyperplasia by downregulation of ROR*γ*t and upregulation of Foxp3 expression, inhibition of IL-17 and promotion of TGF-*β* expression, inhibition of proliferation and promotion of apoptosis in T lymphocytes and increased Tregs ratio	Ma et al., 2019 [156]
	Human UC-MSCs	Rat	Intraperitoneal injection of 2 × 10^6^ UC-MSCs	Slow down the progression of disease activity and reversal of arthritic processes along with triggering of joint tissue repair mechanisms	Vohra et al., 2020 [157]
	Human UC-MSC-sEVs	Rat	ND	Ameliorate arthritis and inhibit synovial hyperplasia in a dose-dependent manner by inhibiting T lymphocyte proliferation and promoting their apoptosis, decreasing Th17 cell proportion and increasing that of Tregs, decreasing serum IL-17, and enhanced IL-10 and TGF-*β* expression, decreasing ROR*γ*t and increased FOXP3 expression	Xu et al., 2021 [158]
Cartilage defects	Human WJ- ECM	Rabbit	1 × 10^6^ rabbit chondrocytes seeded in decellularized WJ-ECM scaffold inserted into the cartilage defects	All defects were filled completely with repaired tissue, and most of which were hyaline cartilage compared to WJ-ECM alone in which the defects filled partially with repaired tissue	Zhao et al., 2018 [159]
	Human WJ-MSCs	Goat	1 × 10^6^ WJ-MSCs seeded in ACECM-oriented scaffold implanted into the articular cartilage defect	The WJ-MSCs-ACECM scaffold complex achieved better quality repair and regeneration of hyaline cartilage compared to microfracture (predominant clinical treatment strategy for damaged cartilage)	Zhang et al., 2018 [160]
	Human WJ-MSCs	Goat	1 × 10^7^ WJ-MSCs and pACs mixed in 3 ratios: 100:0, 0:100 and 50:50 and seeded into ACECM-oriented scaffolds implanted into the articular cartilage defect	50:50 ratio was more similar to native cartilage and better integrated with the surrounding tissue, more abundant cartilage-specific content and significantly higher mechanical strength, no significant joint effusion or bone marrow edema signal. WJ-MSCs possessed low immunogenicity and escaped destruction by the immune system	Zhang et al., 2020 [161]
	Human UC-MSCs-Exosomes	Rabbit	Intra-articular injection of 1 × 10^10^ mL^−1^ of 2D or 3D culture in hollow-fiber bioreactor of UC-MSCs exosomes	Enhanced gross appearance and attenuated cartilage defect; 3D-cultured exosomes showed a superior therapeutic effect	Yan et al., 2020 [162]
	Human UC-MSCs	Rat	WJ/CS composite scaffold loaded with UC-MSCs implanted into the articular cartilage defect	The composite scaffold loaded with UC-MSCs repaired cartilage defects better than did the WJ scaffold loaded with UC-MSCs. Both the scaffold and UC-MSCs showed low immunogenicity	Li et al., 2021 [163]
Osteochondral defects	Rabbit UC-MSCs	Rabbit	PLGA scaffold with a continuous gradient transition between TGF-*β*1 and BMP-2 seeded with 3 × 10^5^ UC-MSCs implanted into articular osteochondral defect	Beneficial effect for bone and cartilage regeneration	Domer et al., 2012 [164]
	Human WJ-MSCs	Rabbit	3 × 10^7^ undifferentiated or chondrogenically induced WJ-MSCs seeded in ECM of swine cartilage-derived scaffolds	Tissues repair observed over 16 months, with a hyaline-like neocartilage layer and regenerated subchondral bone. No immune rejection. WJ-MSCs were superior to those differentiated	Liu et al., 2017 [165]
	Human WJ-MSCs exosomes	Rat and Rabbit	Rat: 25 *μ*g/mL of WJ-MSC exosomes injected in joint cavity (5 times, every 7 days) Rabbit: ACECM scaffold implanted into osteochondral defect with 25 *μ*g/mL of WJ-MSCs exosomes injected in joint cavity, 5 times every 7 days	WJ-MSC exosomes enhance the effect of the ACECM scaffold and promote osteochondral regeneration, regulate the microenvironment of the articular cavity promoting the polarization of macrophages toward the M2 phenotype and inhibiting the inflammatory response. WJ-MSC exosomes contain many miRNAs that can promote the regeneration of hyaline cartilage	Jiang et al., 2021 [166]

ACECM: acellular cartilage extracellular matrix; AM/UC: amniotic membrane/ umbilical cord; CPCs: chondroprogenitor cells; GMs: gelatin microcryogels; GO: graphene oxide; HA: hyaluronic acid; IVD: intervertebral disc; NP: nucleus pulposus; NPCs: NP-like cells; OA: osteoarthritis; pAC: primary cartilage cells; PLGA: poly(D,L-lactic-co-glycolic acid); RA: rheumatoid arthritis; sEVs: small extracellular vesicles; SFC: cartilage superficial; SHED: stem cells derived from human exfoliated deciduous teeth layer cells; WJ/CS: Wharton's jelly and chondroitin sulfate.

**Table 5:** Clinical trials of cartilage repair with UC-MSCs or their secretome.

Pathology	Source	Study design	Delivery mode	Patients (N°)	Results	Ref.
OA	Human UC-MSCs	Randomized, double-blind, controlled phase I/II	Intra-articular injection of 20 × 10^6^ UC-MSCs once or twice vs. HA injection	29	Double injection group showed significant amelioration of pain and disability at 6 and 12 months of follow-up compared to HA group. No severe adverse events were reported.	Matas et al., 2019 [167]
	Human UC-MSCs	Open-label, single arm, phase I/II	Injection of 10 × 10^6^ UC-MSCs in 2 mL secretome + 2 mL HA	29	Significant reduction of the pain and greatest improvement in knee function after 6th-month follow-up.	Dilogo et al., 2020 [168]
	AM/UC particulate	Single-center, investigator-initiated, retrospective study	Inta-articular injection of 100 mg of AM/UC particulate	42	Significant clinical improvement of pain and function in patients with moderate to severe knee OA, with the potential to delay total knee replacement for up to 12 months	Mead et al., 2020 [169]
RA	Human UC-MSCs	Prospective phase I/II study	Intravenous injection of 2 × 10^7^ UC-MSCs	64	Lower levels of serological markers ESR, CRP, RF at 1 and 3 years and anti-CCP at 3 years after treatment. Decrease of health and joint function indexes 1 and 3 years after treatment.	Wang et al., 2019 [170]
	Human UC-MSCs	Phase I/II study	Intravenous drip of 4 × 10^7^ UC-MSCs and intravenous injection of 24 mg of cervus and cucumis peptides	119	Significant reduction of serological markers ESR, CRP, RF, and anti-CCP and improvement of health index and joint function index 1 year after treatment	Qi et al., 2020 [171]
	Human UC-MSCs	Randomized, controlled phase 1/2	Intravenous infusion of 1 × 10^6^ cells/kg of body weight with or without a single intramuscular infusion of 1 million IU of IFN-*γ*	63	Efficacy and ACR20 response rates attained in 53.3% patients with UC-MSCs alone and in 93.3% patients with UC-MSCs combined with IFN-*γ* at 3-month follow-up. No new or unexpected safety issues in 1-year follow-up	He et al., 2020 [172]

ACR20: American College of Rheumatology 20; AM/UC: amniotic membrane/umbilical cord; CCP: cyclic citrullinated peptide (CCP) antibody; CRP: C-reactive protein; ESR: the erythrocyte sedimentation rate; HA: hyaluronic acid; OA: osteoarthritis; RA: rheumatoid arthritis; RF: rheumatoid factor.

## References

[1] Krishnan Y., Grodzinsky A. J. (2018). Cartilage diseases. *Matrix Biology*.

[2] Cieza A., Causey K., Kamenov K., Hanson S. W., Chatterji S., Vos T. (2021). Global estimates of the need for rehabilitation based on the Global Burden of Disease study 2019: a systematic analysis for the Global Burden of Disease Study 2019. *The Lancet*.

[3] Li Y. P., Wei X. C., Zhou J. M., Wei L. (2013). The age-related changes in cartilage and osteoarthritis. *BioMed Research International*.

[4] Arden N., Nevitt M. (2006). Osteoarthritis: epidemiology. *Best Practice & Research. Clinical Rheumatology*.

[5] Hopman W. M., Harrison M. B., Coo H., Friedberg E., Buchanan M., VanDenKerkhof E. G. (2009). Associations between chronic disease, age and physical and mental health status. *Chronic Diseases in Canada*.

[6] Umlauf D., Frank S., Pap T., Bertrand J. (2010). Cartilage biology, pathology, and repair. *Cellular and Molecular Life Sciences*.

[7] O’Neill T. W., Felson D. T. (2018). Mechanisms of osteoarthritis (OA) pain. *Current Osteoporosis Reports*.

[8] Reid M. C., Shengelia R., Parker S. J. (2012). Pharmacologic management of osteoarthritis-related pain in older adults. *HSS Journal*.

[9] Orth P., Rey-Rico A., Venkatesan J. K., Madry H., Cucchiarini M. (2014). Current perspectives in stem cell research for knee cartilage repair. *Stem Cells Cloning*.

[10] Troyer D. L., Weiss M. L. (2008). Concise review: Wharton’s jelly-derived cells are a primitive stromal cell population. *Stem Cells*.

[11] Davies J. E., Walker J. T., Keating A. (2017). Concise review: Wharton's jelly: the rich, but enigmatic, source of mesenchymal stromal cells. *Stem Cells Translational Medicine*.

[12] Liu S., Hou K. D., Yuan M. (2014). Characteristics of mesenchymal stem cells derived from Wharton's jelly of human umbilical cord and for fabrication of non-scaffold tissue-engineered cartilage. *Journal of Bioscience and Bioengineering*.

[13] Yin Y., Li X., He X. T., Wu R. X., Sun H. H., Chen F. M. (2017). Leveraging stem cell homing for therapeutic regeneration. *Journal of Dental Research*.

[14] Eseonu O. I., De Bari C. (2015). Homing of mesenchymal stem cells: mechanistic or stochastic? Implications for targeted delivery in arthritis. *Rheumatology*.

[15] Shen C., Lie P., Miao T. (2015). Conditioned medium from umbilical cord mesenchymal stem cells induces migration and angiogenesis. *Molecular Medicine Reports*.

[16] Kitaori T., Ito H., Schwarz E. M. (2009). Stromal cell-derived factor 1/CXCR4 signaling is critical for the recruitment of mesenchymal stem cells to the fracture site during skeletal repair in a mouse model. *Arthritis and Rheumatism*.

[17] Zhang W., Chen J., Tao J. (2013). The use of type 1 collagen scaffold containing stromal cell-derived factor-1 to create a matrix environment conducive to partial-thickness cartilage defects repair. *Biomaterials*.

[18] Petty J. M., Lenox C. C., Weiss D. J., Poynter M. E., Suratt B. T. (2009). Crosstalk between CXCR4/Stromal Derived Factor-1 and VLA-4/VCAM-1 pathways regulates neutrophil retention in the bone marrow. *The Journal of Immunology*.

[19] Cox D., Brennan M., Moran N. (2010). Integrins as therapeutic targets: lessons and opportunities. *Nature Reviews Drug Discovery*.

[20] Payne N. L., Sun G., McDonald C. (2013). Distinct immunomodulatory and migratory mechanisms underpin the therapeutic potential of human mesenchymal stem cells in autoimmune demyelination. *Cell Transplantation*.

[21] Zou C., Luo Q., Qin J. (2013). Osteopontin promotes mesenchymal stem cell migration and lessens cell stiffness via integrin *β*1, FAK, and ERK pathways. *Cell Biochemistry and Biophysics*.

[22] Lund S. A., Giachelli C. M., Scatena M. (2009). The role of osteopontin in inflammatory processes. *Journal of Cell Communication and Signaling*.

[23] Zhang F., Luo W., Li Y., Gao S., Lei G. (2015). Role of osteopontin in rheumatoid arthritis. *Rheumatology International*.

[24] Cheng C., Gao S., Lei G. (2014). Association of osteopontin with osteoarthritis. *Rheumatology International*.

[25] Schneider R. K., Puellen A., Kramann R. (2010). The osteogenic differentiation of adult bone marrow and perinatal umbilical mesenchymal stem cells and matrix remodelling in three-dimensional collagen scaffolds. *Biomaterials*.

[26] Fu X., Liu G., Halim A., Ju Y., Luo Q., Song G. (2019). Mesenchymal stem cell migration and tissue repair. *Cell*.

[27] Pattappa G., Johnstone B., Zellner J., Docheva D., Angele P. (2019). The importance of physioxia in mesenchymal stem cell chondrogenesis and the mechanisms controlling its response. *International Journal of Molecular Sciences*.

[28] Chow D. C., Wenning L. A., Miller W. M., Papoutsakis E. T. (2001). Modeling pO_2_ Distributions in the Bone Marrow Hematopoietic Compartment. I. Krogh's Model. *Biophysical Journal*.

[29] Bizzarri A., Koehler H., Cajlakovic M. (2006). Continuous oxygen monitoring in subcutaneous adipose tissue using microdialysis. *Analytica Chimica Acta*.

[30] Fischer B., Bavister B. D. (1993). Oxygen-tension in the oviduct and uterus of rhesus-monkeys, hamsters and rabbits. *Journal of Reproduction and Fertility*.

[31] Marmotti A., Mattia S., Castoldi F. (2017). Allogeneic umbilical cord-derived mesenchymal stem cells as a potential source for cartilage and bone regeneration: an in vitro study. *Stem Cells International*.

[32] Lavrentieva A., Majore I., Kasper C., Hass R. (2010). Effects of hypoxic culture conditions on umbilical cord-derived human mesenchymal stem cells. *Cell Communication and Signaling: CCS*.

[33] Nekanti U., Dastidar S., Venugopal P., Totey S., Ta M. (2010). Increased proliferation and analysis of differential gene expression in human Wharton's jelly-derived mesenchymal stromal cells under hypoxia. *International Journal of Biological Sciences*.

[34] Russo E., Lee J. Y., Nguyen H. (2020). Energy metabolism analysis of three different mesenchymal stem cell populations of umbilical cord under normal and pathologic conditions. *Stem Cell Reviews and Reports*.

[35] Kaneko Y., Lee J. Y., Tajiri N. (2020). Translating intracarotid artery transplantation of bone marrow-derived NCS-01 cells for ischemic stroke: behavioral and histological readouts and mechanistic insights into stem cell therapy. *Stem Cells Translational Medicine*.

[36] Neal E. G., Liska M. G., Lippert T. (2018). An update on intracerebral stem cell grafts. *Expert Review of Neurotherapeutics*.

[37] Cozene B., Russo E., Anzalone R., La Rocca G., Borlongan C. (2021). Mitochondrial activity of human umbilical cord mesenchymal stem cells. *Brain Circulation*.

[38] Russo E., Lippert T., Tuazon J. P., Borlongan C. V. (2018). Advancing stem cells: new therapeutic strategies for treating central nervous system disorders. *Brain Circulation*.

[39] Sobolewski K., Małkowski A., Bańkowski E., Jaworski S. (2005). Wharton's jelly as a reservoir of peptide growth factors. *Placenta*.

[40] Qin L., Beier F. (2019). EGFR Signaling: friend or foe for cartilage?. *JBMR Plus*.

[41] Long D. L., Ulici V., Chubinskaya S., Loeser R. F. (2015). Heparin-binding epidermal growth factor-like growth factor (HB-EGF) is increased in osteoarthritis and regulates chondrocyte catabolic and anabolic activities. *Osteoarthritis and Cartilage*.

[42] Zhang X., Liu S., Wang Z. (2021). Implanted 3D gelatin microcryogel enables low-dose cell therapy for osteoarthritis by preserving the viability and function of umbilical cord MSCs. *Chemical Engineering Journal*.

[43] Tonomura H., Nagae M., Takatori R., Ishibashi H., Itsuji T., Takahashi K. (2020). The potential role of hepatocyte growth factor in degenerative disorders of the synovial joint and spine. *International Journal of Molecular Sciences*.

[44] Wang Y., Yuan M., Guo Q., Lu S., Peng J. (2015). Mesenchymal stem cells for treating articular cartilage defects and osteoarthritis. *Cell Transplantation*.

[45] Petrenko Y., Vackova I., Kekulova K. (2020). A comparative analysis of multipotent mesenchymal stromal cells derived from different sources, with a focus on neuroregenerative potential. *Scientific Reports*.

[46] Sulpice E., Ding S., Muscatelli-Groux B. (2009). Cross-talk between the VEGF-A and HGF signalling pathways in endothelial cells. *Biology of the Cell*.

[47] Bobick B. E., Chen F. H., Le A. M., Tuan R. S. (2009). Regulation of the chondrogenic phenotype in culture. *Birth Defects Research Part C: Embryo Today: Reviews*.

[48] Tao Y., Zhou X., Liang C. (2015). TGF-*β*3 and IGF-1 synergy ameliorates nucleus pulposus mesenchymal stem cell differentiation towards the nucleus pulposus cell type through MAPK/ERK signaling. *Growth Factors*.

[49] Palka J., Bańkowski E., Jaworski S. (2000). An accumulation of IGF-I and IGF-binding proteins in human umbilical cord. *Molecular and Cellular Biochemistry*.

[50] La Rocca G., Lo Iacono M., Corsello T., Corrao S., Farina F., Anzalone R. (2013). Human Wharton's jelly mesenchymal stem cells maintain the expression of key immunomodulatory molecules when subjected to osteogenic, adipogenic and chondrogenic differentiation in vitro: new perspectives for cellular therapy. *Current Stem Cell Research & Therapy*.

[51] Wang W., Rigueur D., Lyons K. M. (2014). TGF*β* signaling in cartilage development and maintenance. *Birth Defects Research Part C: Embryo Today: Reviews*.

[52] Patil A. S., Sable R. B., Kothari R. M. (2011). An update on transforming growth factor-*β* (TGF-*β*): sources, types, functions and clinical applicability for cartilage/bone healing. *Journal of Cellular Physiology*.

[53] Liu G. Y., Xu Y., Li Y., Wang L. H., Liu Y. J., Zhu D. (2013). Secreted galectin-3 as a possible biomarker for the immunomodulatory potential of human umbilical cord mesenchymal stromal cells. *Cytotherapy*.

[54] Donders R., Bogie J. F., Ravanidis S. (2018). Human Wharton's jelly-derived stem cells display a distinct immunomodulatory and proregenerative transcriptional signature compared to bone marrow-derived stem cells. *Stem Cells and Development*.

[55] Copland I. B., Adamson S. L., Post M., Lye S. J., Caniggia I. (2002). TGF-*β*3 Expression During Umbilical Cord Development and its Alteration in Pre- eclampsia. *Placenta*.

[56] Deng Z. H., Li Y. S., Gao X., Lei G. H., Huard J. (2018). Bone morphogenetic proteins for articular cartilage regeneration. *Osteoarthritis and Cartilage*.

[57] Scarfì S. (2016). Use of bone morphogenetic proteins in mesenchymal stem cell stimulation of cartilage and bone repair. *World Journal of Stem Cells*.

[58] Kosinski M., Figiel-Dabrowska A., Lech W. (2020). Bone defect repair using a bone substitute supported by mesenchymal stem cells derived from the umbilical cord. *Stem Cells International*.

[59] Choi J., Bae T., Byambasuren N. (2020). CRISPR-Cpf1 Activation of Endogenous _BMP4_ Gene for Osteogenic Differentiation of Umbilical-Cord-Derived Mesenchymal Stem Cells. *Molecular Therapy - Methods & Clinical Development*.

[60] Guo D. B., Zhu X. Q., Li Q. Q. (2018). Efficacy and mechanisms underlying the effects of allogeneic umbilical cord mesenchymal stem cell transplantation on acute radiation injury in tree shrews. *Cytotechnology*.

[61] Zeng J., Wang F., Mao M. (2016). Co-culture of fibroblast-like synoviocytes with umbilical cord-mesenchymal stem cells inhibits expression of pro-inflammatory proteins, induces apoptosis and promotes chondrogenesis. *Molecular Medicine Reports*.

[62] Wang Q., Xu L., Willumeit-Römer R., Luthringer-Feyerabend B. J. (2020). Macrophage-derived oncostatin M/bone morphogenetic protein 6 in response to Mg-based materials influences pro-osteogenic activity of human umbilical cord perivascular cells. *Acta Biomaterialia*.

[63] Frenkel S. R., Saadeh P. B., Mehrara B. J. (2000). Transforming growth factor beta superfamily members: role in cartilage modeling. *Plastic and Reconstructive Surgery*.

[64] Tuan R. S., Chen A. F., Klatt B. A. (2013). Cartilage regeneration. *The Journal of the American Academy of Orthopaedic Surgeons*.

[65] Kangari P., Talaei-Khozani T., Razeghian-Jahromi I., Razmkhah M. (2020). Mesenchymal stem cells: amazing remedies for bone and cartilage defects. *Stem Cell Research & Therapy*.

[66] Mauro A., Buscemi M., Gerbino A. (2010). Immunohistochemical and transcriptional expression of matrix metalloproteinases in full-term human umbilical cord and human umbilical vein endothelial cells. *Journal of Molecular Histology*.

[67] Lo Iacono M., Russo E., Anzalone R. (2018). Wharton's jelly mesenchymal stromal cells support the expansion of cord blood-derived CD34+Cells mimicking a hematopoietic niche in a direct cell-cell contact culture system. *Cell Transplantation*.

[68] Gupta A., el-Amin S. F., Levy H. J., Sze-Tu R., Ibim S. E., Maffulli N. (2020). Umbilical cord-derived Wharton's jelly for regenerative medicine applications. *Journal of Orthopaedic Surgery and Research*.

[69] Trapani M., La Rocca G., Parolini O. (2016). Chapter 6. The immunomodulatory features of mesenchymal stromal cells derived from Wharton’s jelly, amniotic membrane, and chorionic villi: in vitro and in vivo data. *Placenta: The Tree of Life*.

[70] la Rocca G., Anzalone R., Corrao S. (2009). Isolation and characterization of Oct-4+/HLA-G+ mesenchymal stem cells from human umbilical cord matrix: differentiation potential and detection of new markers. *Histochemistry and Cell Biology*.

[71] La Rocca G., Corrao S., Lo Iacono M., Corsello T., Farina F., Anzalone R. (2012). Novel immunomodulatory markers expressed by human WJ-MSC: an updated review in regenerative and reparative medicine. *The Open Tissue Engineering and Regenerative Medicine Journal*.

[72] Anzalone R., Lo Iacono M., Loria T. (2011). Wharton's jelly mesenchymal stem cells as candidates for beta cells regeneration: extending the differentiative and immunomodulatory benefits of adult mesenchymal stem cells for the treatment of type 1 diabetes. *Stem Cell Reviews and Reports*.

[73] Corsello T., Amico G., Corrao S. (2019). Wharton's jelly mesenchymal stromal cells from human umbilical cord: a close-up on immunomodulatory molecules featured in situ and in vitro. *Stem Cell Reviews and Reports*.

[74] Wang Q., Yang Q., Wang Z. (2016). Comparative analysis of human mesenchymal stem cells from fetal-bone marrow, adipose tissue, and Warton's jelly as sources of cell immunomodulatory therapy. *Human Vaccines & Immunotherapeutics*.

[75] Najar M., Raicevic G., Boufker H. I. (2010). Mesenchymal stromal cells use PGE2 to modulate activation and proliferation of lymphocyte subsets: combined comparison of adipose tissue, Wharton's Jelly and bone marrow sources. *Cellular Immunology*.

[76] Anzalone R., Iacono M. L., Corrao S. (2010). New emerging potentials for human Wharton’s jelly mesenchymal stem cells: immunological features and hepatocyte-like differentiative capacity. *Stem Cells and Development*.

[77] di Nicola M., Carlo-Stella C., Magni M. (2002). Human bone marrow stromal cells suppress T-lymphocyte proliferation induced by cellular or nonspecific mitogenic stimuli. *Blood*.

[78] Ma L., Zhou Z., Zhang D. (2012). Immunosuppressive function of mesenchymal stem cells from human umbilical cord matrix in immune thrombocytopenia patients. *Thrombosis and Haemostasis*.

[79] Li W., Zhang Q., Wang M. (2013). Macrophages are involved in the protective role of human umbilical cord- derived stromal cells in renal ischemia-reperfusion injury. *Stem Cell Research*.

[80] Zhao X., Zhao Y., Sun X., Xing Y., Wang X., Yang Q. (2020). Immunomodulation of MSCs and MSC-derived extracellular vesicles in osteoarthritis. *Frontiers in Bioengineering and Biotechnology*.

[81] Tong W., Zhang X., Zhang Q. (2020). Multiple umbilical cord-derived MSCs administrations attenuate rat osteoarthritis progression via preserving articular cartilage superficial layer cells and inhibiting synovitis. *Journal of Orthopaedic Translation*.

[82] Zhang Q., Xiang E., Rao W. (2021). Intra-articular injection of human umbilical cord mesenchymal stem cells ameliorates monosodium iodoacetate-induced osteoarthritis in rats by inhibiting cartilage degradation and inflammation. *Bone & Joint Research*.

[83] Kim H., Yang G., Park J., Choi J., Kang E., Lee B. K. (2019). Therapeutic effect of mesenchymal stem cells derived from human umbilical cord in rabbit temporomandibular joint model of osteoarthritis. *Scientific Reports*.

[84] Voisin C., Cauchois G., Reppel L. (2020). Are the immune properties of mesenchymal stem cells from Wharton's jelly maintained during chondrogenic differentiation?. *Journal of Clinical Medicine*.

[85] Giannini D., Antonucci M., Petrelli F., Bilia S., Alunno A., Puxeddu I. (2020). One year in review 2020: pathogenesis of rheumatoid arthritis. *Clinical and Experimental Rheumatology*.

[86] Zhao C., Zhang L., Kong W. (2015). Umbilical cord-derived mesenchymal stem cells inhibit cadherin-11 expression by fibroblast-like synoviocytes in rheumatoid arthritis. *Journal of Immunology Research*.

[87] Hildner F., Wolbank S., Redl H., van Griensven M., Peterbauer A. (2010). How chondrogenic are human umbilical cord matrix cells? A comparison to adipose-derived stem cells. *Journal of Tissue Engineering and Regenerative Medicine*.

[88] Ruan D., Zhang Y., Wang D. (2012). Differentiation of human Wharton's jelly cells toward nucleus pulposus-like cells after coculture with nucleus pulposus CellsIn vitro. *Tissue Engineering. Part A*.

[89] Chon B. H., Lee E. J., Jing L., Setton L. A., Chen J. (2013). Human umbilical cord mesenchymal stromal cells exhibit immature nucleus pulposus cell phenotype in a laminin-rich pseudo-three-dimensional culture system. *Stem Cell Research & Therapy*.

[90] Caballero M., Skancke M. D., Halevi A. E. (2013). Effects of connective tissue growth factor on the regulation of elastogenesis in human umbilical cord-derived mesenchymal stem cells. *Annals of Plastic Surgery*.

[91] Marmotti A., Mattia S., Bruzzone M. (2012). Minced umbilical cord fragments as a source of cells for orthopaedic tissue engineering: an in vitro study. *Stem Cells International*.

[92] Danišovič L., Boháč M., Zamborský R. (2016). Comparative analysis of mesenchymal stromal cells from different tissue sources in respect to articular cartilage tissue engineering. *General Physiology and Biophysics*.

[93] Wu H., Zeng X., Yu J. (2017). Comparison of nucleus pulposus stem/progenitor cells isolated from degenerated intervertebral discs with umbilical cord derived mesenchymal stem cells. *Experimental Cell Research*.

[94] Bailey M. M., Wang L., Bode C. J., Mitchell K. E., Detamore M. S. (2007). A comparison of human umbilical cord matrix stem cells and temporomandibular joint condylar chondrocytes for tissue engineering temporomandibular joint condylar cartilage. *Tissue Engineering*.

[95] Islam A., Urbarova I., Bruun J. A., Martinez-Zubiaurre I. (2019). Large-scale secretome analyses unveil the superior immunosuppressive phenotype of umbilical cord stromal cells as compared to other adult mesenchymal stromal cells. *European Cells & Materials*.

[96] Zhang Y., Liu S., Guo W. (2019). Coculture of hWJMSCs and pACs in oriented scaffold enhances hyaline cartilage regeneration in vitro. *Stem Cells International*.

[97] Li X., Liang Y., Xu X. (2019). Cell-to-cell culture inhibits dedifferentiation of chondrocytes and induces differentiation of human umbilical cord-derived mesenchymal stem cells. *BioMed Research International*.

[98] Han Z., Zhang Y., Gao L., Jiang S., Ruan D. (2018). Human Wharton's jelly cells activate degenerative nucleus pulposus cells in vitro. *Tissue Engineering. Part A*.

[99] Toh W. S., Lee E. H., Cao T. (2011). Potential of human embryonic stem cells in cartilage tissue engineering and regenerative medicine. *Stem Cell Reviews and Reports*.

[100] Castro-Viñuelas R., Sanjurjo-Rodríguez C., Piñeiro-Ramil M. (2018). Induced pluripotent stem cells for cartilage repair: current status and future perspectives. *European Cells & Materials*.

[101] Hwang J. J., Rim Y. A., Nam Y., Ju J. H. (2021). Recent developments in clinical applications of mesenchymal stem cells in the treatment of rheumatoid arthritis and osteoarthritis. *Frontiers in Immunology*.

[102] Jyothi Prasanna S., Sowmya Jahnavi V. (2011). Wharton's jelly mesenchymal stem cells as off-the-shelf cellular therapeutics: a closer look into their regenerative and immunomodulatory properties. *The Open Tissue Engineering and Regenerative Medicine Journal*.

[103] Khan W. S., Tew S. R., Adesida A. B., Hardingham T. E. (2008). Human infrapatellar fat pad-derived stem cells express the pericyte marker 3G5 and show enhanced chondrogenesis after expansion in fibroblast growth factor-2. *Arthritis Research & Therapy*.

[104] Hatakeyama A., Uchida S., Utsunomiya H. (2017). Isolation and characterization of synovial mesenchymal stem cell derived from hip joints: a comparative analysis with a matched control knee group. *Stem Cells International*.

[105] Amable P. R., Teixeira M. V., Carias R. B., Granjeiro J. M., Borojevic R. (2014). Protein synthesis and secretion in human mesenchymal cells derived from bone marrow, adipose tissue and Wharton’s jelly. *Stem Cell Research & Therapy*.

[106] Raicevic G., Najar M., Stamatopoulos B. (2011). The source of human mesenchymal stromal cells influences their TLR profile as well as their functional properties. *Cellular Immunology*.

[107] Balasubramanian S., Venugopal P., Sundarraj S., Zakaria Z., Majumdar A. S., Ta M. (2012). Comparison of chemokine and receptor gene expression between Wharton's jelly and bone marrow-derived mesenchymal stromal cells. *Cytotherapy*.

[108] Mckinnirey F., Herbert B., Vesey G., McCracken S. (2021). Immune modulation via adipose derived mesenchymal stem cells is driven by donor sex in vitro. *Scientific Reports*.

[109] Mohamed-Ahmed S., Fristad I., Lie S. A. (2018). Adipose-derived and bone marrow mesenchymal stem cells: a donor-matched comparison. *Stem Cell Research & Therapy*.

[110] Mead B., Logan A., Berry M., Leadbeater W., Scheven B. A. (2014). Paracrine-mediated neuroprotection and neuritogenesis of axotomised retinal ganglion cells by human dental pulp stem cells: comparison with human bone marrow and adipose-derived mesenchymal stem cells. *PLoS One*.

[111] Batsali A. K., Pontikoglou C., Koutroulakis D. (2017). Differential expression of cell cycle and WNT pathway-related genes accounts for differences in the growth and differentiation potential of Wharton’s jelly and bone marrow-derived mesenchymal stem cells. *Stem Cell Research & Therapy*.

[112] Li C. Y., Wu X. Y., Tong J. B. (2015). Comparative analysis of human mesenchymal stem cells from bone marrow and adipose tissue under xeno-free conditions for cell therapy. *Stem Cell Research & Therapy*.

[113] Shin S., Lee J., Kwon Y. (2021). Comparative proteomic analysis of the mesenchymal stem cells secretome from adipose, bone marrow, placenta and Wharton's jelly. *International Journal of Molecular Sciences*.

[114] Heirani-Tabasi A., Toosi S., Mirahmadi M. (2017). Chemokine receptors expression in MSCs: comparative analysis in different sources and passages. *Tissue engineering and regenerative medicine*.

[115] Najar M., Raicevic G., Jebbawi F. (2012). Characterization and functionality of the CD200-CD200R system during mesenchymal stromal cell interactions with T-lymphocytes. *Immunology Letters*.

[116] Karaöz E., Demircan P. C., Erman G., Güngörürler E., Sarıboyaci A. E. (2017). Comparative analyses of immunosuppressive characteristics of bone-marrow, Wharton’s jelly, and adipose tissue-derived human mesenchymal stem cells. *Turkish Journal of Haematology*.

[117] Purandare B., Teklemariam T., Zhao L., Hantash B. M. (2014). Temporal HLA profiling and immunomodulatory effects of human adult bone marrow- and adipose-derived mesenchymal stem cells. *Regenerative Medicine*.

[118] Mennan C., Garcia J., Roberts S., Hulme C., Wright K. (2019). A comprehensive characterisation of large-scale expanded human bone marrow and umbilical cord mesenchymal stem cells. *Stem Cell Research & Therapy*.

[119] Camilleri E. T., Gustafson M. P., Dudakovic A. (2016). Identification and validation of multiple cell surface markers of clinical-grade adipose-derived mesenchymal stromal cells as novel release criteria for good manufacturing practice-compliant production. *Stem Cell Research & Therapy*.

[120] Pappa A. K., Caballero M., Dennis R. G. (2014). Biochemical properties of tissue-engineered cartilage. *The Journal of Craniofacial Surgery*.

[121] Cashion A. T., Caballero M., Halevi A., Pappa A., Dennis R. G., van Aalst J. A. (2014). Programmable mechanobioreactor for exploration of the effects of periodic vibratory stimulus on mesenchymal stem cell differentiation. *BioResearch Open Access*.

[122] Remya N. S., Nair P. D. (2016). Mechanoresponsiveness of human umbilical cord mesenchymal stem cells in in vitro chondrogenesis-a comparative study with growth factor induction. *Journal of Biomedical Materials Research. Part A*.

[123] Zhao L., Detamore M. S. (2010). Chondrogenic differentiation of stem cells in human umbilical cord stroma with PGA and PLLA scaffolds. *Journal of Biomedical Science and Engineering*.

[124] Fong C. Y., Subramanian A., Gauthaman K. (2012). Human umbilical cord Wharton's jelly stem cells undergo enhanced chondrogenic differentiation when grown on nanofibrous scaffolds and in a sequential two-stage culture medium environment. *Stem Cell Reviews and Reports*.

[125] Chen X., Zhang F., He X. (2013). Chondrogenic differentiation of umbilical cord-derived mesenchymal stem cells in type I collagen-hydrogel for cartilage engineering. *Injury*.

[126] Nirmal R. S., Nair P. D. (2013). Significance of soluble growth factors in the chondrogenic response of human umbilical cord matrix stem cells in a porous three dimensional scaffold. *European Cells & Materials*.

[127] Sridharan B., Lin S. M., Hwu A. T., Laflin A. D., Detamore M. S. (2015). Stem cells in aggregate form to enhance chondrogenesis in hydrogels. *PLoS One*.

[128] Jaipaew J., Wangkulangkul P., Meesane J., Raungrut P., Puttawibul P. (2016). Mimicked cartilage scaffolds of silk fibroin/hyaluronic acid with stem cells for osteoarthritis surgery: morphological, mechanical, and physical clues. *Materials Science & Engineering. C, Materials for Biological Applications*.

[129] Wang J., Sun B., Tian L. (2017). Evaluation of the potential of rhTGF- *β*3 encapsulated P(LLA-CL)/collagen nanofibers for tracheal cartilage regeneration using mesenchymal stems cells derived from Wharton's jelly of human umbilical cord. *Materials Science & Engineering. C, Materials for Biological Applications*.

[130] Aleksander-Konert E., Paduszyński P., Zajdel A., Dzierżewicz Z., Wilczok A. (2016). In vitro chondrogenesis of Wharton's jelly mesenchymal stem cells in hyaluronic acid-based hydrogels. *Cellular & Molecular Biology Letters*.

[131] Zhang W., Yang J., Zhu Y. (2019). Extracellular matrix derived by human umbilical cord-deposited mesenchymal stem cells accelerates chondrocyte proliferation and differentiation potential in vitro. *Cell and Tissue Banking*.

[132] Wang L., Tran I., Seshareddy K., Weiss M. L., Detamore M. S. (2009). A comparison of human bone marrow-derived mesenchymal stem cells and human umbilical cord-derived mesenchymal stromal cells for cartilage tissue engineering. *Tissue Engineering. Part A*.

[133] Wang L., Seshareddy K., Weiss M. L., Detamore M. S. (2009). Effect of initial seeding density on human umbilical cord mesenchymal stromal cells for fibrocartilage tissue engineering. *Tissue Engineering. Part A*.

[134] Wang L., Zhao L., Detamore M. S. (2011). Human umbilical cord mesenchymal stromal cells in a sandwich approach for osteochondral tissue engineering. *Journal of Tissue Engineering and Regenerative Medicine*.

[135] Qi L., Wang R., Shi Q., Yuan M., Jin M., Li D. (2019). Umbilical cord mesenchymal stem cell conditioned medium restored the expression of collagen II and aggrecan in nucleus pulposus mesenchymal stem cells exposed to high glucose. *Journal of Bone and Mineral Metabolism*.

[136] Penolazzi L., Pozzobon M., Bergamin L. S. (2020). Extracellular matrix from decellularized Wharton's jelly improves the behavior of cells from degenerated intervertebral disc. *Frontiers in Bioengineering and Biotechnology*.

[137] Leckie S. K., Sowa G. A., Bechara B. P. (2013). Injection of human umbilical tissue-derived cells into the nucleus pulposus alters the course of intervertebral disc degeneration in vivo. *The Spine Journal*.

[138] Beeravolu N., Brougham J., Khan I., McKee C., Perez-Cruet M., Chaudhry G. R. (2018). Human umbilical cord derivatives regenerate intervertebral disc. *Journal of Tissue Engineering and Regenerative Medicine*.

[139] Perez-Cruet M., Beeravolu N., McKee C. (2019). Potential of human nucleus pulposus-like cells derived from umbilical cord to treat degenerative disc disease. *Neurosurgery*.

[140] Ekram S., Khalid S., Bashir I., Salim A., Khan I. (2021). Human umbilical cord-derived mesenchymal stem cells and their chondroprogenitor derivatives reduced pain and inflammation signaling and promote regeneration in a rat intervertebral disc degeneration model. *Molecular and Cellular Biochemistry*.

[141] Saulnier N., Viguier E., Perrier-Groult E. (2015). Intra-articular administration of xenogeneic neonatal mesenchymal stromal cells early after meniscal injury down-regulates metalloproteinase gene expression in synovium and prevents cartilage degradation in a rabbit model of osteoarthritis. *Osteoarthritis and Cartilage*.

[142] Raines A. L., Shih M., Chua L., Su C., Tseng S. C., O'Connell J. (2017). Efficacy of particulate amniotic membrane and umbilical cord tissues in attenuating cartilage destruction in an osteoarthritis model. *Tissue Engineering. Part A*.

[143] Chang H. S., Wu K. C., Liu H. W., Chu T. Y., Ding D. C. (2018). Human umbilical cord-derived mesenchymal stem cells reduce monosodium iodoacetate-induced apoptosis in cartilage. *Ci Ji Yi Xue Za Zhi*.

[144] Zhang B. Y., Wang B. Y., Li S. C. (2018). Evaluation of the curative effect of umbilical cord mesenchymal stem cell therapy for knee arthritis in dogs using imaging technology. *Stem Cells International*.

[145] Kim S. E., Pozzi A., Yeh J. C. (2019). Intra-articular umbilical cord derived mesenchymal stem cell therapy for chronic elbow osteoarthritis in dogs: a Double-Blinded, Placebo-Controlled Clinical Trial. *Frontiers in veterinary science*.

[146] Geng Y., Chen J., Alahdal M. (2020). Intra-articular injection of hUC-MSCs expressing miR-140-5p induces cartilage self-repairing in the rat osteoarthritis. *Journal of Bone and Mineral Metabolism*.

[147] Wu K. C., Chang Y. H., Liu H. W., Ding D. C. (2019). Transplanting human umbilical cord mesenchymal stem cells and hyaluronate hydrogel repairs cartilage of osteoarthritis in the minipig model. *Ci Ji Yi Xue Za Zhi*.

[148] Magri C., Schramme M., Febre M. (2019). Comparison of efficacy and safety of single versus repeated intra-articular injection of allogeneic neonatal mesenchymal stem cells for treatment of osteoarthritis of the metacarpophalangeal/metatarsophalangeal joint in horses: a clinical pilot study. *PLoS One*.

[149] Perry J., McCarthy H. S., Bou-Gharios G. (2020). Injected human umbilical cord-derived mesenchymal stromal cells do not appear to elicit an inflammatory response in a murine model of osteoarthritis. *Osteoarthritis and cartilage open*.

[150] Xing D., Wu J., Wang B. (2020). Intra-articular delivery of umbilical cord-derived mesenchymal stem cells temporarily retard the progression of osteoarthritis in a rat model. *International Journal of Rheumatic Diseases*.

[151] Wang X. D., Wan X. C., Liu A. F., Li R., Wei Q. (2021). Effects of umbilical cord mesenchymal stem cells loaded with graphene oxide granular lubrication on cytokine levels in animal models of knee osteoarthritis. *International Orthopaedics*.

[152] Liu Y., Mu R., Wang S. (2010). Therapeutic potential of human umbilical cord mesenchymal stem cells in the treatment of rheumatoid arthritis. *Arthritis Research & Therapy*.

[153] Wu C. C., Wu T. C., Liu F. L., Sytwu H. K., Chang D. M. (2012). TNF-*α* inhibitor reverse the effects of human umbilical cord-derived stem cells on experimental arthritis by increasing immunosuppression. *Cellular Immunology*.

[154] Gu J., Gu W., Lin C. (2015). Human umbilical cord mesenchymal stem cells improve the immune-associated inflammatory and prothrombotic state in collagen type-II-induced arthritic rats. *Molecular Medicine Reports*.

[155] Zhang Q., Li Q., Zhu J. (2019). Comparison of therapeutic effects of different mesenchymal stem cells on rheumatoid arthritis in mice. *PeerJ*.

[156] Ma D., Xu K., Zhang G. (2019). Immunomodulatory effect of human umbilical cord mesenchymal stem cells on T lymphocytes in rheumatoid arthritis. *International Immunopharmacology*.

[157] Vohra M., Sharma A., Bagga R., Arora S. K. (2020). Human umbilical cord-derived mesenchymal stem cells induce tissue repair and regeneration in collagen-induced arthritis in rats. *Journal of Clinical and Translational Research*.

[158] Xu K., Ma D., Zhang G. (2021). Human umbilical cord mesenchymal stem cell-derived small extracellular vesicles ameliorate collagen-induced arthritis via immunomodulatory T lymphocytes. *Molecular Immunology*.

[159] Zhao P., Liu S., Bai Y. (2018). hWJECM-derived oriented scaffolds with autologous chondrocytes for rabbit cartilage defect repairing. *Tissue Engineering. Part A*.

[160] Zhang Y., Liu S., Guo W. (2018). Human umbilical cord Wharton's jelly mesenchymal stem cells combined with an acellular cartilage extracellular matrix scaffold improve cartilage repair compared with microfracture in a caprine model. *Osteoarthritis and Cartilage*.

[161] Zhang Y., Hao C., Guo W. (2020). Co-culture of hWJMSCs and pACs in double biomimetic ACECM oriented scaffold enhances mechanical properties and accelerates articular cartilage regeneration in a caprine model. *Stem Cell Research & Therapy*.

[162] Yan L., Wu X. (2020). Exosomes produced from 3D cultures of umbilical cord mesenchymal stem cells in a hollow-fiber bioreactor show improved osteochondral regeneration activity. *Cell Biology and Toxicology*.

[163] Li Z., Bi Y., Wu Q. (2021). A composite scaffold of Wharton's jelly and chondroitin sulphate loaded with human umbilical cord mesenchymal stem cells repairs articular cartilage defects in rat knee. *Journal of Materials Science. Materials in Medicine*.

[164] Dormer N. H., Singh M., Zhao L., Mohan N., Berkland C. J., Detamore M. S. (2012). Osteochondral interface regeneration of the rabbit knee with macroscopic gradients of bioactive signals. *Journal of Biomedical Materials Research. Part A*.

[165] Liu S., Jia Y., Yuan M. (2017). Repair of Osteochondral Defects Using Human Umbilical Cord Wharton’s Jelly- Derived Mesenchymal Stem Cells in a Rabbit Model. *BioMed Research International*.

[166] Jiang S., Tian G., Yang Z. (2021). Enhancement of acellular cartilage matrix scaffold by Wharton's jelly mesenchymal stem cell-derived exosomes to promote osteochondral regeneration. *Bioactive Materials*.

[167] Matas J., Orrego M., Amenabar D. (2019). Umbilical cord-derived mesenchymal stromal cells (MSCs) for knee osteoarthritis: repeated MSC dosing is superior to a single MSC dose and to hyaluronic acid in a controlled randomized phase I/II trial. *Stem Cells Translational Medicine*.

[168] Dilogo I. H., Canintika A. F., Hanitya A. L., Pawitan J. A., Liem I. K., Pandelaki J. (2020). Umbilical cord-derived mesenchymal stem cells for treating osteoarthritis of the knee: a single-arm, open-label study. *European Journal of Orthopaedic Surgery and Traumatology*.

[169] Mead O. G., Mead L. P. (2020). Intra-articular injection of amniotic membrane and umbilical cord particulate for the management of moderate to severe knee osteoarthritis. *Orthopedic Research and Reviews*.

[170] Wang L., Huang S., Li S. (2019). Efficacy and safety of umbilical cord mesenchymal stem cell therapy for rheumatoid arthritis patients: a prospective phase I/II study. *Drug Design, Development and Therapy*.

[171] Qi T., Gao H., Dang Y., Huang S., Peng M. (2020). Cervus and cucumis peptides combined umbilical cord mesenchymal stem cells therapy for rheumatoid arthritis. *Medicine (Baltimore)*.

[172] He X., Yang Y., Yao M. (2020). Combination of human umbilical cord mesenchymal stem (stromal) cell transplantation with IFN-*γ* treatment synergistically improves the clinical outcomes of patients with rheumatoid arthritis. *Annals of the Rheumatic Diseases*.

